# Dynamic DNA binding licenses a repair factor to bypass roadblocks in search of DNA lesions

**DOI:** 10.1038/ncomms10607

**Published:** 2016-02-03

**Authors:** Maxwell W. Brown, Yoori Kim, Gregory M. Williams, John D. Huck, Jennifer A. Surtees, Ilya J. Finkelstein

**Affiliations:** 1 Department of Molecular Biosciences, Institute for Cellular and Molecular Biology, The University of Texas at Austin, Austin, Texas 78712, USA; 2 Department of Biochemistry, School of Medicine and Biomedical Sciences, State University of New York at Buffalo, Buffalo, New York 14214, USA; 3 Center for Systems and Synthetic Biology, The University of Texas at Austin, Austin, Texas 78712, USA

## Abstract

DNA-binding proteins search for specific targets via facilitated diffusion along a crowded genome. However, little is known about how crowded DNA modulates facilitated diffusion and target recognition. Here we use DNA curtains and single-molecule fluorescence imaging to investigate how Msh2–Msh3, a eukaryotic mismatch repair complex, navigates on crowded DNA. Msh2–Msh3 hops over nucleosomes and other protein roadblocks, but maintains sufficient contact with DNA to recognize a single lesion. In contrast, Msh2–Msh6 slides without hopping and is largely blocked by protein roadblocks. Remarkably, the Msh3-specific mispair-binding domain (MBD) licences a chimeric Msh2–Msh6(3MBD) to bypass nucleosomes. Our studies contrast how Msh2–Msh3 and Msh2–Msh6 navigate on a crowded genome and suggest how Msh2–Msh3 locates DNA lesions outside of replication-coupled repair. These results also provide insights into how DNA repair factors search for DNA lesions in the context of chromatin.

## Abstract (web summary)

DNA-binding proteins need to scan the genome to find their targets though how this occurs in the crowded nuclear environment is poorly understood. Here the authors use single-molecular fluorescence to show the repair complex Msh2-Msh3 can hop over blocks that impede the sliding complex Msh2-Msh6.

## Graphical abstract

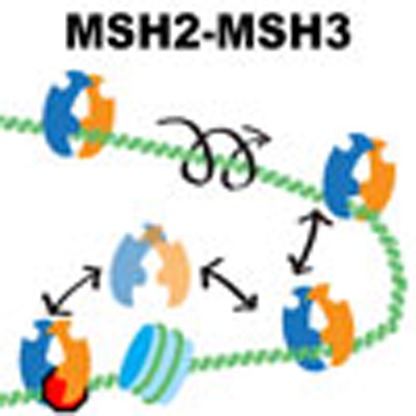

DNA-binding proteins must rapidly locate specific sites amidst a vast pool of non-specific DNA. To accelerate the search process, these proteins reduce the total search space by employing a combination of three-dimensional (3D) diffusion through the nucleus and facilitated one-dimensional (1D) diffusion along the DNA^1^. During 1D diffusion, proteins can either slide along the helical pitch of the DNA backbone, or can transiently dissociate and associate with the DNA via a series of microscopic hops. Both sliding and hopping have been observed *in vitro* via single-molecule and ensemble biochemistry approaches, and have also been inferred via single-molecule imaging in live cells^2^,^3^,^4^,^5^,^6^. Indeed, 1D-facilitated diffusion is a common feature of nearly all proteins that scan both DNA^1^,^2^,^3^ and RNA^7^,^8^ for specific sequences, structures or lesions.

In the eukaryotic nucleus, these proteins must also navigate on chromatin crowded with nucleosomes and other DNA-binding proteins. While the role of nucleosomes and other roadblocks in modulating facilitated diffusion has been considered computationally^9^,^10^, there is scant direct evidence that diffusing proteins can bypass nucleosomes and other DNA-bound roadblocks while still recognizing specific DNA sequences or structures. To experimentally address this question, we investigated facilitated diffusion by yeast Msh2–Msh3 and Msh2–Msh6, two heterodimeric MutS homologue (Msh) complexes that participate in the first step of eukaryotic mismatch repair (MMR)^11^,^12^. Both Msh complexes form sliding clamps on DNA and scan the genome for a partially overlapping but distinct spectrum of DNA mismatches and other extrahelical lesions^13^,^14^,^15^. Once a lesion is found, the Msh complex binds and recruits downstream protein factors to initiate repair. *In vitro* studies have established that Msh2–Msh6 can scan naked DNA for lesions via 1D facilitated diffusion along the DNA track^14^,^15^,^16^. However, both yeast and human Msh2–Msh6 diffusion is blocked by nucleosomes *in vitro*^17^,^18^. This led to a model in which Msh2–Msh6 mainly scans newly synthesized DNA at the replication fork, which is transiently nucleosome-free^19^,^20^,^21^,^22^.

How Msh2–Msh3 scans a crowded DNA remains unexplored and the *in vivo* interactions between Msh2–Msh3 and the replication fork are less clear. Msh2–Msh3 is also implicated in other genome maintenance pathways that occur outside of replication-coupled MMR, suggesting that it must scan DNA in the context of nucleosomes^21^,^23^,^24^,^25^,^26^. Thus Msh2–Msh3 may employ a unique strategy for navigating protein-bound DNA. Here we use single-molecule fluorescence microscopy to reveal that Msh2–Msh3 scans DNA via a facilitated diffusion mechanism comprised of both 1D sliding and microscopic hopping. Msh2–Msh3's DNA interactions are sufficiently dynamic to allow the bypass of nucleosomes and other protein obstacles, while still allowing the complex to recognize a single DNA lesion. In contrast, Msh2–Msh6 does not hop on DNA and is largely blocked by nucleosomes. Remarkably, a chimeric version of Msh2–Msh6 that encodes the Msh3 mispair-binding domain (MBD) imparts roadblock bypass activity to Msh2–Msh6. Thus the Msh3 MBD is sufficient to license Msh complex hopping. Our studies contrast how Msh2–Msh3 and Msh2–Msh6 navigate a crowded genome and suggest how Msh2–Msh3 functions outside of replication-coupled repair. More broadly, we provide a model for how dynamic fluctuations within DNA-encircling protein domains may facilitate bypass of other protein roadblocks during 1D-facilitated diffusion.

## Results

### Visualizing Msh2–Msh3 sliding on DNA curtains

We investigated how Msh2–Msh3 slides on DNA by directly monitoring the protein's movement via total internal reflection fluorescence microscopy of fluorescently labelled Msh2–Msh3. Yeast Msh2–Msh3 with a hemagglutinin (HA) epitope tag on the Msh2 subunit was overexpressed and purified from yeast cells (Supplementary Fig. 1). To fluorescently label Msh2–Msh3, we conjugated the protein with anti-HA antibody-coupled quantum dots (QDs). Gel shift and ATPase assays indicated that the QD-tagged Msh2–Msh3 retained biochemical activities similar to wild-type protein and remained responsive to specific DNA templates (Supplementary Fig. 1). These data indicate that the QD does not compromise communication between the DNA-binding and ATPase domains of Msh2–Msh3. This epitope-labelling strategy has also been used successfully with yeast Msh2–Msh6 (refs 17, 27).

**Figure 1:**
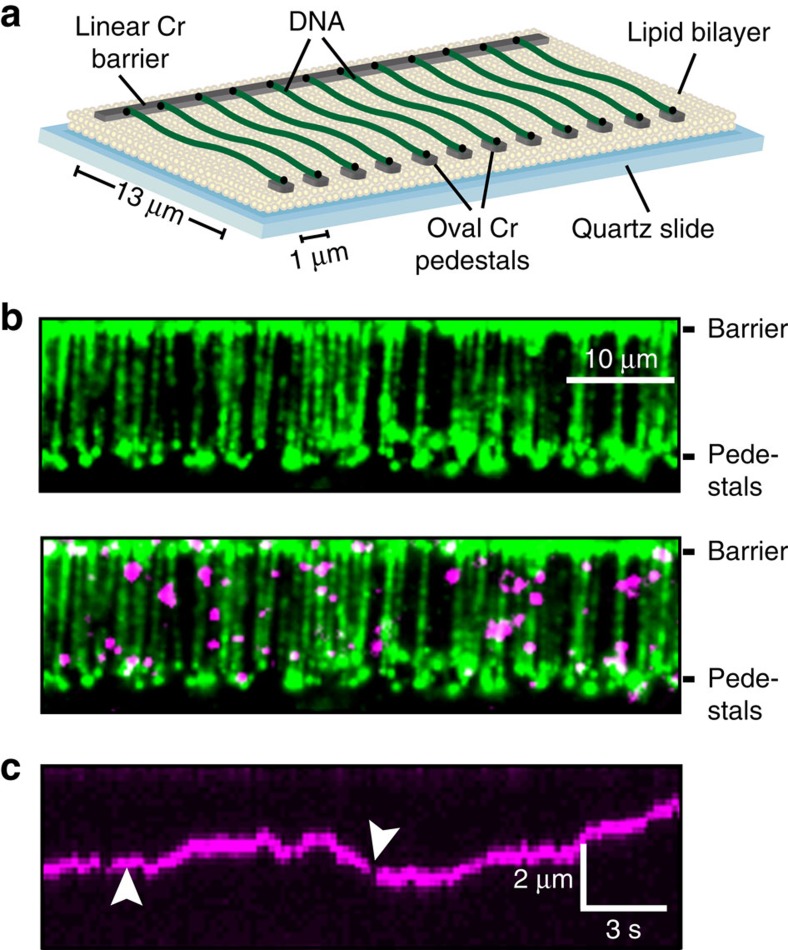
Visualizing protein diffusion on aligned arrays of DNA molecules. (**a**) An illustration of the DNA curtains assay (Supplementary Information). A quartz microscope slide is fabricated with an alternating pattern of linear chromium (Cr) diffusion barriers and oval pedestals (∼30 nm tall; 13 μm separation). The pedestals are coated with anti-digoxigenin antibodies. The flowcell surface is passivated with a fluid lipid bilayer (∼5 nm tall), and DNA (from λ-phage, 48,502 bp) is affixed to the bilayer via a biotin-streptavidin linkage. Buffer flow is used to organize DNA molecules at the linear diffusion barriers and the free DNA end is immobilized at the Cr pedestals via a digoxigenin–antibody interaction. DNA molecules that are tethered at both ends remain extended when buffer flow is turned off. (**b**) A double-tethered DNA curtain. DNA is stained with YOYO-1, a fluorescent intercalating dye (green; top). Quantum dot (QD)-conjugated Msh2–Msh3 binds specifically to the DNA molecules (magenta; bottom). We did not observe any QD signal when Msh2–Msh3 was omitted from the incubation, or when Msh2–Msh3 was incubated with an unconjugated QD. YOYO-1 was omitted from subsequent experiments because it can cause laser-induced DNA damage. Scale bar: 10 μm. (**c**) Kymograph of a single diffusing Msh2–Msh3 protein. QDs blinking (white arrows) indicates that these traces arise from single fluorescent particles.

We used a high-throughput DNA curtain assay for assembling precisely positioned arrays of DNA molecules on the surface of a microfluidic flowcell (Fig. 1a)^17^,^28^,^29^. In this double-tethered DNA curtains assay, a microscope slide was passivated with a fluid lipid bilayer. λ-phage DNA (48,502 bp long) was deposited on the surface of the slide and tethered between lithographically fabricated chromium (Cr) diffusion barriers. One end of the DNA molecule was biotinylated and affixed to a fluid lipid bilayer via a biotin–streptavidin linkage. The second DNA end was labelled with digoxigenin (DIG) and captured at an anti-DIG antibody-coated Cr pedestal positioned 13 μm away from the linear diffusion barrier^28^. Double-tethered DNA remains in an extended state, allowing us to image Msh2–Msh3 in the absence of any hydrodynamic force^29^. Following DNA curtain assembly, we injected fluorescently labelled Msh2–Msh3 into the flowcells, and observed protein co-localization with the extended DNA molecules (Fig. 1b). DNA-bound QDs were only detected when Msh2–Msh3 and anti-HA-conjugated QDs were pre-incubated before injection into the flowcell. Pre-incubating Msh2–Msh3 with unconjugated QDs did not result in any DNA-bound QDs^30^. In our typical imaging buffer conditions (40 mM Tris-HCl pH 8, 25–150 mM NaCl, 2 mM MgCl_2_, 2 mM DTT, 0.2 mg ml^−1^ BSA), we observed intermittent fluorescent emission (blinking) from the diffraction-limited fluorescent particles. Blinking is an intrinsic property of single QDs and is partially suppressed by including 1–2 mM DTT in the imaging buffer^31^. As two QDs are unlikely to blink simultaneously, these blinking events indicate that our fluorescent trajectories are from individual fluorescent QD-protein particles (Fig. 1c). We conclude that Msh2–Msh3 is singly labelled via its HA epitope tag and that the HA-tagged Msh2–Msh3 specifically binds DNA.

### Msh2–Msh3 scans DNA via hopping and 1D diffusion

**Figure 2:**
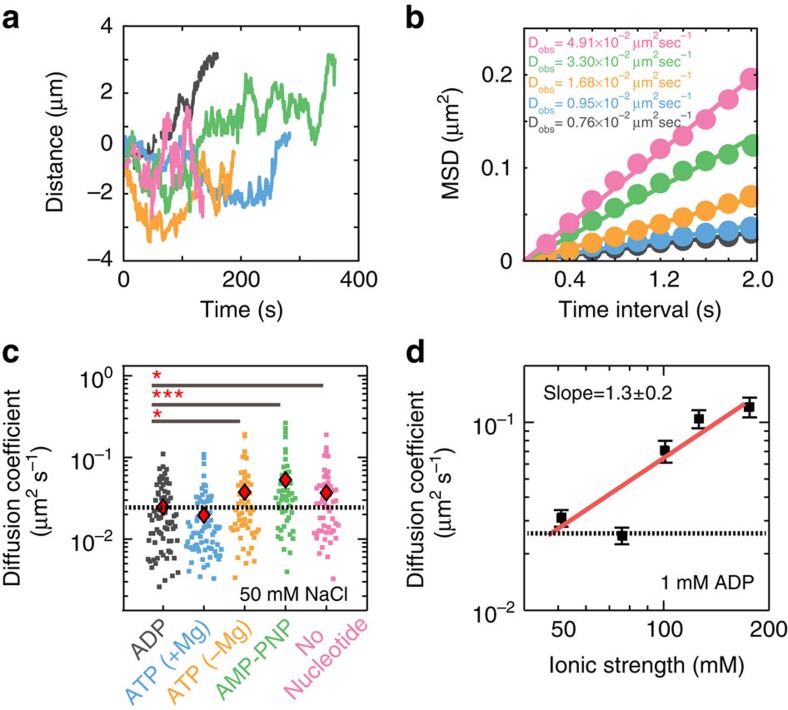
Msh2–Msh3 scans DNA via one-dimensional (1D) sliding. (**a**) Representative traces of diffusing Msh2–Msh3 molecules with 1 mM of the indicated nucleotide and 50 mM NaCl in the imaging buffer (black: ADP; blue: ATP; orange: ATP–Mg^+2^; green: AMP–PNP; pink: no nucleotide). (**b**) The trajectories in **a** were used to calculate mean squared displacements (MSD) and the MSDs for each molecule were used to obtain an apparent 1D diffusion coefficient (black: ADP; blue: ATP; orange: ATP-Mg^+2^; green: AMP-PNP; pink: no nucleotide). Solid lines indicate linear fits through the MSD points. (**c**) Diffusion coefficients for at least 50 molecules in each nucleotide state (with 50 mM NaCl). Red diamonds indicate the mean of the distribution. *P value <0.05 and ***P value <0.001. There is a statistically significant twofold increase in the mean diffusion confidents with non-hydrolyzable nucleotides (*P* values: 2.5 × 10^−2^, 1.4 × 10^−4^, and 1.2 × 10^−2^ for ATP–Mg^+2^, AMP–PNP, and no nucleotide, respectively). Dashed line: theoretical limit for sliding with rotation along the DNA backbone. Supplementary Table 1 summarizes the means, s.d., and additional *P* values for each nucleotide condition. (**d**) Msh2–Msh3 diffusion coefficients increase with higher ionic strength. Error bars represent the s.e.m. A linear fit to the log–log plot has a slope of 1.3±0.2, suggesting ∼1.5 charge–charge interactions between Msh2–Msh3 and DNA are disrupted at increasing ionic strengths. Dashed line: theoretical limit for sliding with rotation along the DNA backbone. Each data point represents the mean of at least 47 diffusing particles, and all results are summarized in Supplementary Table 2.

Fluorescently labelled Msh2–Msh3 readily associated with the double-tethered DNA curtains and >90% (*n*=584) of the DNA-bound molecules exhibited sliding behaviour in the absence of buffer flow (Fig. 1c). These observations are consistent with Msh2–Msh3's high affinity for homoduplex DNA in both gel-shift and surface plasmon resonance based assays^32^,^33^,^34^,^35^. The time-dependent fluorescent signals were fit to a two-dimensional Gaussian^36^ function and the resulting trajectories were used to analyze the movement of Msh2–Msh3 along the DNA molecule (Fig. 2a). Msh2–Msh3 trajectories had a net displacement of zero base pairs, as would be expected for molecules that are undergoing thermally driven diffusion (Supplementary Fig. 2). To characterize how Msh2–Msh3 scans the DNA, we computed the mean-squared displacements (MSD, examples in Fig. 2b) and diffusion coefficients (Fig. 2c) for each sliding molecule. The range of observed diffusion coefficients is consistent with a scanning mode where Msh2–Msh3 partially tracks the helical twist of the DNA duplex (see below)^3^,^27^,^37^,^38^,^39^,^40^.

Msh2 and Msh3 each harbour non-equivalent Walker-type ATP hydrolysis sites, and ADP to ATP exchange is a key feature of mismatch release by all Msh proteins^41^,^42^,^43^,^44^,^45^,^46^,^35^. To probe the impact of nucleotides on Msh2–Msh3 interactions with DNA, we varied the nucleotides that were included in the flow buffer and measured their effect on diffusion (Fig. 2c). Msh2–Msh3 diffusion coefficients were nucleotide dependent, increasing approximately twofold from 1 mM ADP (mean=0.025±0.021 μm^2^ s^−1^; *n*=72, range indicates s.d.) to 1 mM AMP–PNP (mean=0.053±0.058 μm^2^ s^−1^; *n*=50) or ATP in the absence of Mg^+2^ (mean=0.038±0.039 μm^2^ s^−1^; *n*=56). In contrast, the diffusion coefficients of yeast Msh2–Msh6 were nucleotide independent^27^. A complete summary of these results is provided in Supplementary Table 1. Biochemical studies with both human and yeast proteins have suggested that the Msh2 and Msh3 subunits differ in their nucleotide binding and hydrolysis activities when the Msh2–Msh3 complex is bound to homoduplex DNA^35^,^42^,^47^. Here we show that ADP and ATP also alter the interactions of the yeast Msh2–Msh3 complex with homoduplex DNA, presumably through conformational changes that are communicated from the ATPase domains to the DNA-binding domain. The lowest diffusion coefficients were with ADP, suggesting that the ADP-bound state interacts most strongly with the DNA and is thus poised for lesion recognition.

Proteins can scan non-specific DNA via 1D sliding, hopping and/or intersegmental transfer. During 1D sliding, the protein retains continuous contact with the DNA, while hopping is characterized by a series of correlated microscopic detachment and reattachment events. Intersegmental transfer can occur when a protein transfers between two DNA sites by directly binding both sides of a DNA loop. Intersegmental transfer is unlikely in our experiments because the DNA molecules are kept in an extended state, precluding looping. Furthermore, intersegmental transfer over distances larger than ∼1 kb would appear as punctate trajectories with rapid protein re-localization between two distal DNA sites. Observation of over 300 diffusing Msh2–Msh3 molecules did not reveal any such discontinuous trajectories on extended DNA molecules.

To differentiate between sliding and hopping, we measured Msh2–Msh3 diffusion coefficients at increasing ionic strengths (Fig. 2d and Supplementary Table 2). A higher ionic strength increases electrostatic screening between a protein and DNA. This reduces the fraction of time that a protein is in contact with the DNA and results in increased diffusion coefficients at higher ionic strengths. This approach has recently been used to differentiate between sliding and hopping for a variety of DNA-binding proteins^40^,^48^,^49^. Msh2–Msh3 diffusion coefficient increased fourfold, from 0.031±0.027 μm^2^ s^−1^ at an ionic strength of 51 mM (*n*=47) to 0.12±0.14 μm^2^ s^−1^ (*n*=49; *P* value: 7.5 × 10^−7^) at an ionic strength of 176 mM (Fig. 2d and Supplementary Table 2). For a protein that mainly interacts with DNA via electrostatic interactions, the diffusion coefficient reports on *K*_D_, the microscopic dissociation constant^37^,^50^. The number of charge–charge interactions between the protein and DNA can be estimated from the slope of the log(D_1D_) versus log(*I*), where *I* is the total ionic strength. The slope of the fit to the data in Fig. 2d was 1.3±0.2 (root-mean-square error), which corresponds to 1.5±0.2 (root-mean-square error) screened charges with an ion condensation parameter of 0.88 for double-stranded DNA^50^. In contrast, Msh2–Msh6 diffusion was not salt-dependent^27^ and MutS diffusion was weakly salt dependent (0.23±0.01 screened charges)^45^. We conclude that Msh2–Msh3 hops while diffusing, and that the hopping is facilitated by weak electrostatic contacts between Msh2–Msh3 and DNA.

**Figure 3:**
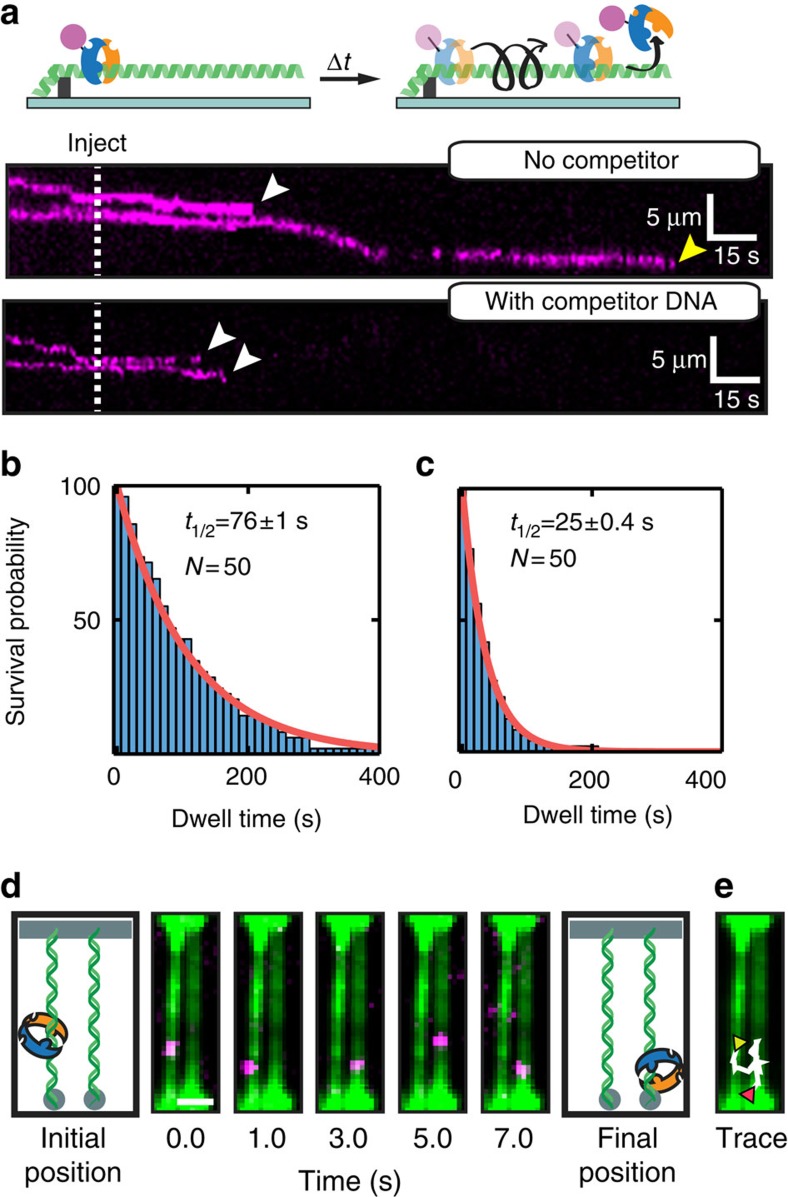
Msh2–Msh3 transiently dissociates from DNA during 1D sliding. (**a**) Cartoon illustration (top) and a kymograph (bottom) of Msh2–Msh3 dissociating from a single-tethered DNA molecule. In the absence of competitor DNA (mock injection), Msh2–Msh3 slides along the DNA and dissociates from both internal sites (white arrow) and from free DNA ends (yellow arrow). Msh2–Msh3 dissociates from DNA curtains more rapidly after competitor DNA is injected in the flowcell (dashed line). Quantification of the Msh2–Msh3 lifetimes (**b**) without or (**c**) with competitor DNA. Lifetimes are fit to a single exponential decay and the half-lives±s.e. are reported in the panels. (**d**) Msh2–Msh3 (magenta) can transfer between adjacent DNA molecules. Initially, Msh2–Msh3 diffuses on the left DNA molecule. After 3 s, the complex transfers to an adjacent DNA. After the diffusion data was acquired, the DNA molecules were stained with YOYO-1 (green). Scale bar, 2 μm (**e**) A trace of the complete trajectory (white) is superimposed on the locations of the two DNA molecules. The starting and ending points are indicated by yellow and red triangles, respectively.

The MutS DNA-binding clamp can undergo large conformational rearrangements in the absence of DNA^44^,^51^ and when bound to homoduplex DNA^51^. Thus, we reasoned that Msh2–Msh3 may hop on DNA by transiently opening and closing its DNA-binding clamp, which encircles the DNA duplex in co-crystal structures of Muts homologs with DNA^43^,^44^,^52^,^53^. Transient opening of the DNA clamp could allow Msh2 and/or Msh3 to briefly detach from the DNA, while rapid re-closing would prevent the protein from dissociating into solution (Fig. 3a, top panel). This model suggests three testable hypotheses: (i) diffusing Msh2–Msh3 will dissociate from both internal sites (clamp opening) as well as free DNA ends (sliding off the DNA); (ii) Msh2–Msh3 dwell times will be sensitive to the addition of competitor DNA; and (iii) Msh2–Msh3 may hop between two closely positioned DNA molecules. First, we measured the dissociation positions and dwell times of Msh2–Msh3 on single-tethered DNA curtains (Fig. 3a). In this assay, one of the DNA ends is attached to the lipid bilayer surface, while the second end remains free in solution^29^. Continuous buffer flow is used to keep the DNA extended and also biases protein diffusion towards the free DNA end. The availability of a free DNA end allowed us to measure both Msh2–Msh3 dissociation from internal sites and sliding off from the free DNA ends (Fig. 3a). We observed that 80% (*n*=40/50; 50 mM NaCl, 1 mM ADP) of diffusing Msh2–Msh3 molecules dissociated from internal DNA sites, with nearly half of those molecules (*n*=21/40) sliding for at least ∼1 kb before dissociation (Fig. 3a, middle panel). The remaining 20% (*n*=10/50) of the molecules slid off the free DNA end. We also measured the dwell times of Msh2–Msh3 on single-tethered DNA curtains. In the absence of competitor DNA, the Msh2–Msh3 half-life±s.e. was 76±1.0 s (*n*=50; Fig. 3b). The Msh2–Msh3 half-life was reduced threefold (25±0.4 s, *n*=50) after addition of homoduplex competitor DNA (4 μM 39-mer double-stranded oligo; Fig. 3c). In contrast, the half-life of Msh2–Msh6 was not dependent on the addition of competitor DNA (Supplementary Fig. 3 and ref. 27).

We also observed that Msh2–Msh3 could transfer between two closely positioned DNA molecules (Fig. 3d). For these experiments, we assembled high-density double-tethered DNA curtains and analysed regions of the flowcell where two extended DNA molecules were laterally separated by ∼1 μm, the closest spacing between our Cr pedestals. We observed that Msh2–Msh3 complexes could transfer between two such adjacent DNA molecules (Fig. 3d). Here Msh2–Msh3 scans the left DNA molecule, followed by transfer and diffusion on a neighbouring DNA. We observed such transfer events for 46% (*n*=23/50) of diffusing Msh2–Msh3 molecules (with 1 mM ADP, 100 mM NaCl in the imaging buffer). These observations do not stem from binding by two different Msh2–Msh3 complexes because all free enzymes have been flushed out of the flowcell. We also ruled out the possibility that these observations are due to several Msh2–Msh3 proteins per QD by conjugating fewer than one antibody per QD^54^. Msh2–Msh3 can transfer between the two DNA strands via either intersegmental transfer or by hopping. The DNA molecules were fluorescently labelled after the Msh2–Msh3 diffusion traces were acquired, so we cannot unambiguously distinguish between these two mechanisms. Regardless, our results demonstrate that Msh2–Msh3 scans the genome by a combination of hopping and 1D sliding, and that these facilitated diffusion modes are consistent with transient opening of the DNA-binding clamp.

### Diffusing Msh2–Msh3 can bypass protein obstacles on DNA

**Figure 4:**
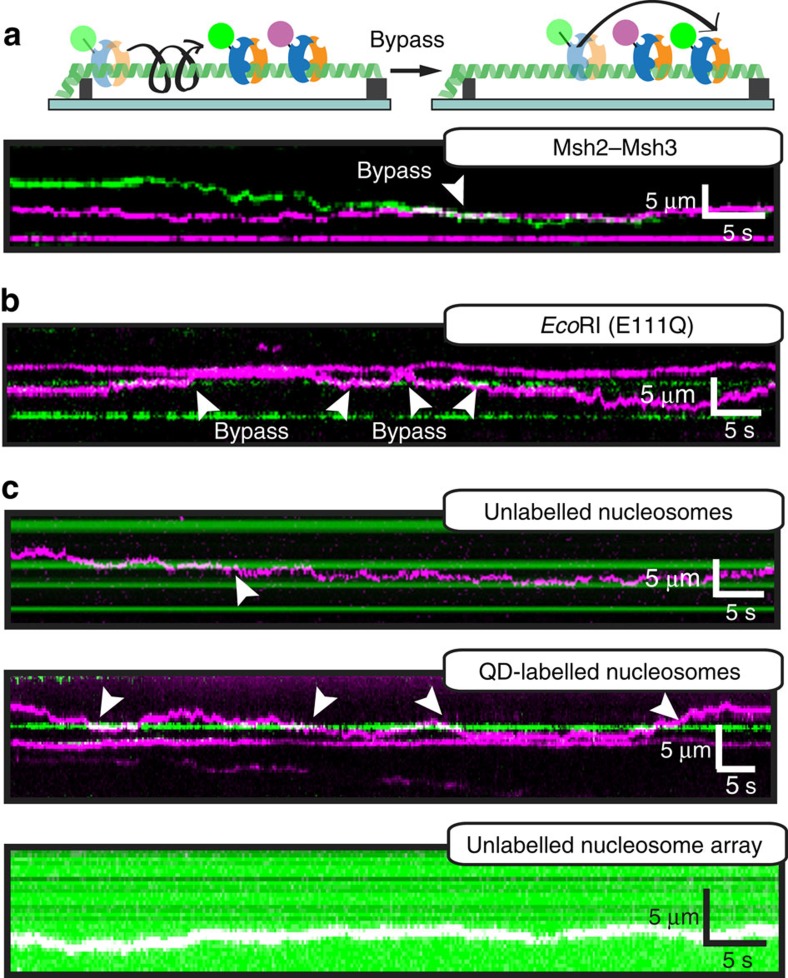
Diffusing Msh2–Msh3 bypasses protein roadblocks. (**a**) Cartoon illustration (top) and kymograph (bottom) of green and magenta Msh2–Msh3 complexes bypassing each other on the same DNA molecule. The bypass events are indicated with white arrowheads. (**b**) Kymograph of Msh2–Msh3 (magenta) bypassing *Eco*RI(E111Q) (green). (**c**) Kymographs of Msh2–Msh3 bypassing unlabelled nucleosomes (top, green) and QD-labeled nucleosomes (middle, green). Msh2–Msh3 also diffuses on dense nucleosome arrays (bottom, green). These arrays appear completely green due to the large quantity of post-labelled nucleosomes. Msh2–Msh3 (magenta) appears as white when co-localized with nucleosomes.

We next tested whether dynamic opening of the Msh2–Msh3 DNA clamp may also facilitate bypass of other DNA-bound proteins that would be encountered during the lesion search process. First, we investigated whether two diffusing Msh2–Msh3 complexes can bypass each other as they slide on the same DNA molecule. For these experiments, two Msh2–Msh3 fractions were each conjugated with spectrally distinct QDs—the first emitted in the green channel (605 nm peak fluorescence emission) and the second in the magenta channel (705 nm emission). The differentially labelled proteins were mixed in a 1:1 ratio and injected into a flowcell with pre-assembled DNA curtains (Fig. 4a). As expected, both species readily diffused on DNA. We observed bypass events when two differentially labelled molecules collided (Fig. 4a), suggesting that one Msh2–Msh3 can bypass a second diffusing molecule on the same DNA strand. Next, we determined whether Msh2–Msh3 also bypasses other protein roadblocks. We used *Eco*RI(E111Q), a hydrolytically defective restriction enzyme that is frequently used as a model protein roadblock^55^. Fluorescent *Eco*RI(E111Q) retains sub-nanomolar binding affinity to the five *Eco*RI sites in our DNA substrate^30^ and has previously been shown to block diffusing MutS^16^. Remarkably, Msh2–Msh3 readily bypassed fluorescent *Eco*RI(E111Q), indicating that it can diffuse past both moving and stationary protein roadblocks (Fig. 4b).

Nucleosomes are the most frequent DNA obstacles that Msh2–Msh3 encounters *in vivo*. To explore how Msh2–Msh3 navigates on a nucleosome-coated DNA track, we purified recombinant histone octamers with an N-terminal triple-FLAG epitope tag on the H2A subunit (Supplementary Fig. 4a). Epitope-labelled and wild-type histone octamers were indistinguishable in gel-based reconstitution assays (Supplementary Fig. 4b). We deposited an average of 4.5±1.7 nucleosomes on the DNA substrate via a salt-dialysis protocol (Supplementary Fig. 4 and Methods). Next, we determined whether Msh2–Msh3 bypassed nucleosomes that are not conjugated with QDs (unlabelled nucleosomes). In these assays, nucleosome-coated DNA was first assembled into DNA curtains after which fluorescent Msh2–Msh3 was flushed into the flowcell. After 10 min of monitoring Msh2–Msh3 diffusion, the experiments were stopped and nucleosomes were labelled *in situ* with fluorescently labelled antibodies. Msh2–Msh3 readily bypassed unlabelled nucleosomes as it diffused on DNA (Fig. 4c, top). We ruled out the possibility that these observations are due to several Msh2–Msh3 proteins per QD by observing hopping with HA antibodies labelled with the much smaller Alexa488 fluorescent probe and also when Msh2–Msh3 was incubated with a large excess of QDs (1:5 protein:QD ratio; Supplementary Fig. 4e). To quantify the nucleosome bypass frequency, we scored all Msh2–Msh3 diffusion trajectories that entered a 750 bp ‘collision zone' centred on a nucleosome. The collision zone was defined as three times the s.d. of our precision in localizing a fluorescent nucleosome. A collision was scored as a bypass event when Msh2–Msh3 diffused from one side of a nucleosome to the other through the collision zone. We observed nucleosome bypass events in 46% of all collisions (53/115 collisions; 58 trajectories). Msh2–Msh3 also bypassed QD-labelled nucleosomes (605 nm emission, ∼10 nm radius^39^), creating a much larger barrier to 1D diffusion (Fig. 4c, middle). Remarkably, the bypass probability was 43% (12/28 collisions; 17 trajectories), nearly identical to unlabelled nucleosome obstacles. Next, we monitored collisions between Msh2–Msh3 and dense nucleosome arrays. For these experiments, DNA curtains were reconstituted at fourfold higher octamer to DNA ratios, such that we could no longer resolve individual nucleosomes. After reconstitution at these increased ratios, we estimated 20 or more nucleosomes per DNA molecule. Surprisingly, 58% (*n*=30/52) of Msh2–Msh3 molecules continued to show diffusive motion on these nucleosome-coated DNA substrates (Fig. 4c, bottom). As nucleotide binding modulated the apparent diffusion coefficient on naked DNA (Fig. 2c), we determined the effect of nucleotide on Msh2–Msh3's ability to bypass nucleosomes (Supplementary Fig. 5). Msh2–Msh3 hopped over nucleosomes in the presence of all nucleotides, but we observed fewer nucleosome bypass events when AMP–PNP (28%, *n*=14/50) or ATP–MgCl_2_ (33%, *n*=16/50) was included in the imaging buffer (Supplementary Fig. 5). These results further support the model that nucleotide binding triggers conformational changes that are transmitted to the DNA clamp domain, ultimately altering Msh2–Msh3 facilitated diffusion on both naked and crowded DNA^35^,^42^,^56^. In contrast, Msh2–Msh6 is rarely able to pass unlabelled nucleosomes, and is completely blocked by QD-conjugated nucleosomes (Supplementary Fig. 6). As with Msh2–Msh3, we confirmed that Alexa488-αHA labelled Msh2–Msh6 was also blocked by nucleosomes, indicating that QDs do not contribute to the observed differences in facilitated diffusion between the two Msh complexes (Supplementary Fig. 6d). These results highlight that Msh2–Msh3, unlike Msh2–Msh6, has the potential to hop over nucleosomes and other protein obstacles as it scans the genome for DNA lesions.

### The Msh3 MBD enables roadblock bypass

**Figure 5:**
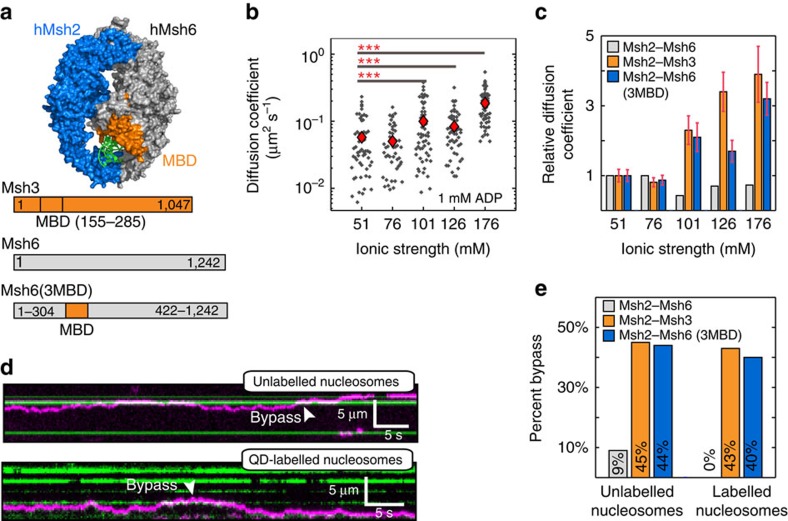
Characterizing the sliding of a chimeric Msh2–Msh6(3MBD). (**a**) Top: hMSH2–MSH6 structure (PDB: 2O8B). Msh2, Msh6 and DNA are shown in blue, grey and green, respectively. The Msh6–MBD is coloured in orange and makes multiple contacts with the DNA. Below: domain map of yeast Msh2–Msh6(3MBD) chimera. (**b**) Msh2–Msh6(3MBD) diffusion coefficients as a function of total ionic strength (*n*⩾50 for each condition). Red diamonds indicate the mean diffusion coefficients. Asterisks indicate ***P value <0.001. Diffusion coefficients increase at higher ionic strengths (see Supplementary Table 3 for *P* values). (**c**) Summary of the relative diffusion coefficients for Msh2–Msh6 (grey, from ref. 27), Msh2–Msh3 (orange, this study) and Msh2–Msh6(3MBD) (blue, this study). Diffusion coefficients for each protein are normalized to their respective values at the lowest ionic strength. Error bars are the s.e.m. (**d**) Kymographs of Msh2–Msh6(3MBD) (magenta) bypassing unlabelled (top, green) and QD-labelled (bottom, green) nucleosomes. (**e**) Quantification of the nucleosome bypass frequencies for each of the three heterodimers (unlabelled nucleosomes: *n*=100 for Msh2–Msh6, Msh2–Msh3, and Msh2–Msh6(3MBD); pre-labelled nucleosomes: *n*=31, 28 and 25 for Msh2–Msh6, Msh2–Msh3 and Msh2–Msh6(3MBD)). The data for Msh2–Msh6 are acquired in this study and agree with a previous study^17^.

Our results highlighted dramatic differences between the scanning modes of Msh2–Msh3 and Msh2–Msh6 on homoduplex and nucleosome-coated DNA. As the Msh2 subunit is present in both heterodimers, we reasoned that the MBDs of the Msh6 and Msh3 subunits may regulate these differences between the two complexes. To test this hypothesis, we characterized a chimeric Msh2–Msh6 in which the Msh6 MBD is swapped for the Msh3 MBD^20^. This Msh2–Msh6(3MBD) chimera (Fig. 5a) partially rescues Msh3-null phenotypes *in vivo* and exhibits increased specificity for Msh2–Msh3-like lesions *in vitro*^20^. We introduced an HA epitope tag into the Msh2 subunit of Msh2–Msh6(3MBD), and assayed the activity of the chimeric protein via ATPase and electrophoretic mobility shift assays (EMSA) (Supplementary Fig. 7). The ATPase activity of the chimeric complex was responsive to DNA both with and without QDs (Supplementary Fig. 7b). In accordance with previous studies, Msh2–Msh6(3MBD) had an increased affinity for a +8 insertion/deletion loop (Supplementary Fig. 7c)^20^. For single-molecule assays, Msh2–Msh6(3MBD) was labelled with QDs, as described for Msh2–Msh3 and Msh2–Msh6. Fluorescent Msh2–Msh6(3MBD) readily bound DNA curtains with 76% (*n*=269) of the molecules showing diffusive trajectories on DNA (Supplementary Fig. 7).

Remarkably, replacing the Msh6 MBD substantially altered the dynamic behaviour of the Msh complex on DNA, more closely resembling Msh2–Msh3 than Msh2–Msh6. Msh2–Msh6(3MBD) diffusion coefficients increased monotonically with increasing ionic strength (Fig. 5b and Supplementary Table 3), indicating that, like Msh2–Msh3, this construct diffuses via a combination of sliding and hopping. Conversely, Msh2–Msh6 did not exhibit this behaviour (Fig. 5c and ref. 27). Furthermore, Msh2–Msh6(3MBD) readily transferred between adjacent DNA molecules (44%, *n*=22/50), and also bypassed nucleosome roadblocks (Fig. 5d). Remarkably, both of these activities occurred with nearly the same frequencies for Msh2–6(3MBD) and Msh2–Msh3 (Fig. 5e). In contrast, only 8% (*n*=4/50) of Msh2–Msh6 molecules transferred between adjacent DNA strands. These results are consistent with structural and functional studies that have shown distinct DNA-binding modes for Msh2–Msh3 and Msh2–Msh6 (refs 43, 57). Our single-molecule data indicate that these structural differences are translated into distinct dynamic behaviours on homoduplex DNA substrates that have important implications for the respective search mechanisms. We conclude that the Msh3 MBD is sufficient to alter the dynamics within the Msh2–Msh6 DNA-binding clamp, which stimulates a combination of facilitated diffusion and roadblock bypass activities of the Msh2–Msh6(3MBD) chimera.

### Msh2–Msh3 bypasses nucleosomes during lesion recognition

**Figure 6:**
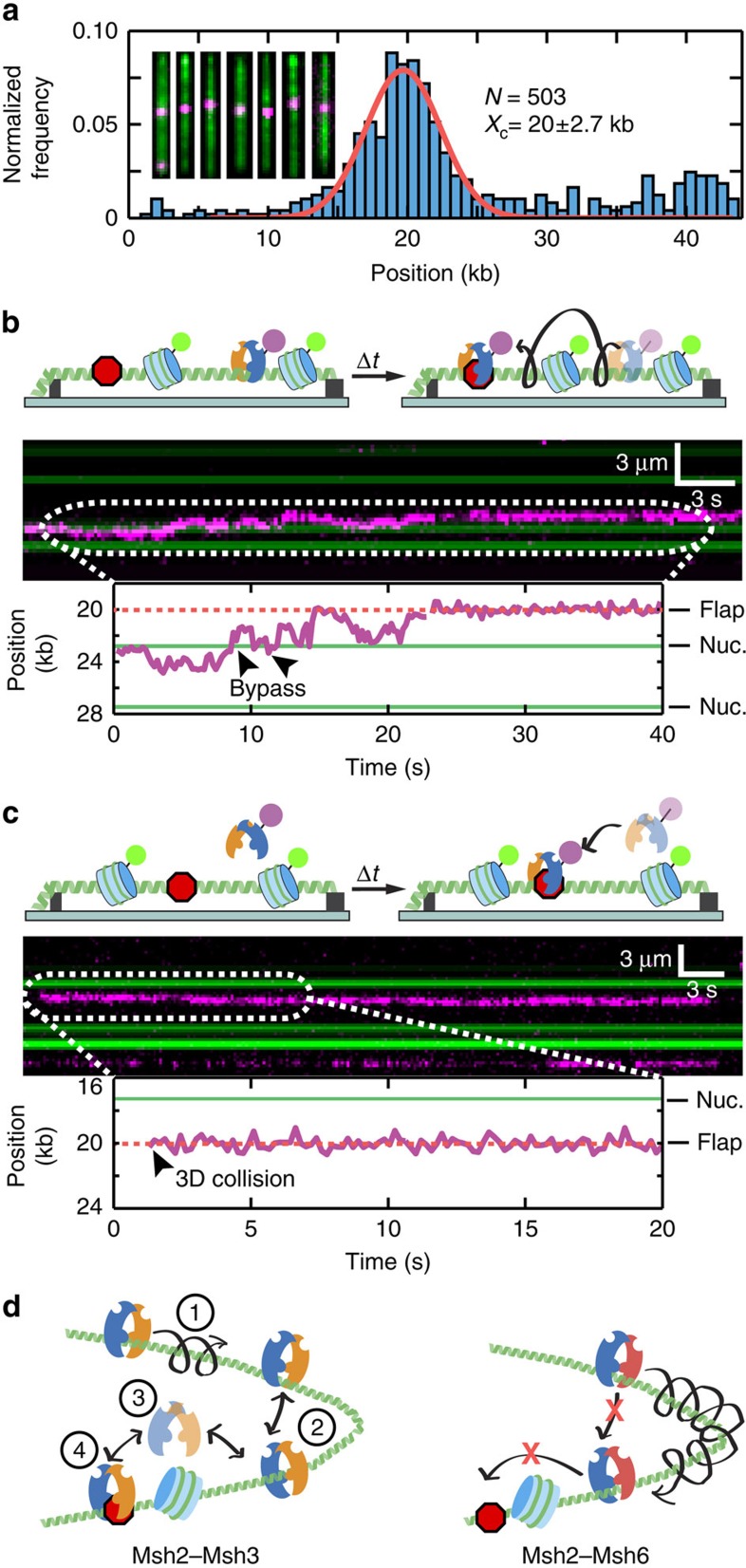
Msh2–Msh3 recognizes DNA lesions via both 1D sliding and 3D collisions. (**a**) Distribution of Msh2–Msh3 molecules on lesion-containing DNA. The red line is a Gaussian fit to the data (*n*=503). The center of the peak corresponds to the expected location of the DNA flap (20 kb from the top DNA barrier). The inset shows seven representative DNA molecules with flap-bound Msh2–Msh3. (**b**) Cartoon illustration (top) and kymograph (middle) of Msh2–Msh3 (magenta) hopping over a nucleosome (post labelled; green) and stopping at a DNA lesion (3′-ssDNA flap; red octagon). The corresponding single-particle trajectory is shown below. The Msh2–Msh3 trajectory is in magenta, the nucleosome position is represented with a solid green line, and the flap position is indicated as a dashed red line (also see Supplementary Fig. 8). (**c**) Cartoon (top), kymograph (middle), and single-particle trajectory of Msh2–Msh3 (magenta) recognizing a 3′-ssDNA flap via 3D collision (bottom). (**d**) A model for how Msh2–Msh3 (left) and Msh2–Msh6 (right) scan DNA to find a lesion. Msh2–Msh3 diffuses via a combination of 1D sliding (1) and hopping (2). Msh2–Msh3 dynamics facilitate transient release from the DNA track and hopping over nucleosomes (3) but still support lesion recognition (red octagon) (4). In contrast, Msh2–Msh6 does not hop on DNA and is blocked by a nucleosome roadblock.

Msh2–Msh3 recognizes and facilitates processing of 3′ single-stranded non-homologous tail DNA structures during single-strand annealing (SSA), which can occur throughout the cell cycle and is not coupled to DNA replication^21^. Msh2–Msh3 may thus need to bypass nucleosomes as it scans the genome to recognize these single-strand DNA (ssDNA) flaps. Therefore, we explored Msh2–Msh3's ability to recognize specific lesions on a nucleosome-coated DNA track. We introduced an 18-nucleotide 3′-ssDNA flap 20 kb downstream of the biotinylated DNA end (Supplementary Fig. 8a and Supplementary Table 4). Over 95% of the DNA molecules incorporated the 3′-ssDNA flap (Supplementary Fig. 8c and Information), and site-specific incorporation was confirmed by both gel assays and single-molecule fluorescence imaging (Supplementary Fig. 8). We next incubated Msh2–Msh3 with the lesion-containing DNA and measured the locations of DNA-bound Msh2–Msh3 complexes (Fig. 6a). The DNA-binding histogram showed a strong enrichment at the lesion, indicating that the complex specifically binds the 3′-ssDNA flap (Fig. 6a). We also observed an increased affinity for 12 nucleotide 5′-ssDNA ends over homoduplex regions, with 9% of the molecules localizing to the vicinity of these free DNA ends in our single-tethered DNA curtain assay (inset, Fig. 6a). These results agree with previous studies that have reported increased affinity of Msh proteins for ss/double-stranded DNA junctions^34^.

Msh2–Msh3 has the potential to recognize lesions by one of two non-exclusive mechanisms: (i) 1D scanning (Fig. 6b) and (ii) 3D collisions (Fig. 6c). To observe lesion recognition, we assembled the lesion-containing DNA substrate into a double-tethered DNA curtain and imaged the lesion-recognition reaction in real-time (with 1 mM ADP, in the absence of buffer flow). We saw evidence of both lesion recognition mechanisms, with 27% of the molecules (*n*=4/15) directly binding the lesion via a 3D collision mechanism (within our ∼300 bp resolution). The remaining 73% of molecules diffused for at least 1 kb along the DNA before stopping at the lesion site. Next, we deposited nucleosomes on the lesion-containing DNA (Fig. 6b). Remarkably, diffusing Msh2–Msh3 could readily bypass a nucleosome en route to binding the 3′-ssDNA flap (Fig. 6b). We limited our analysis to nucleosomes that were at least 1 kb away from the DNA lesion, ensuring that we could resolve both the hopping and lesion recognition events by the same Msh complex. These results show that Msh2–Msh3 can hop over nucleosomes while maintaining its ability to recognize a single DNA lesion.

## Discussion

Msh proteins form sliding clamps on DNA to recognize mismatches and other DNA structures that arise during DNA replication and homologous recombination (HR). We show that Msh2–Msh3 scans DNA via a combination of sliding and hopping, a fundamentally different mechanism than previously reported for prokaryotic MutS and eukaryotic Msh2–Msh6 (refs 27, 45, 51, 58). Hopping is facilitated by rapid opening and closing of the Msh2–Msh3 DNA-binding clamp, as revealed by three lines of evidence: (i) the apparent 1D diffusion coefficient increased ∼fourfold when the total ionic strength is varied from 51 to 176 mM (Fig. 2d); (ii) Msh2–Msh3 dwell times on DNA are dependent on the addition of competitor DNA (Fig. 3b,c); and (iii) Msh2–Msh3 can dynamically transfer between two neighbouring DNA molecules (Fig. 3d). In addition to increasing the apparent diffusion coefficient, hopping permits Msh2–Msh3 to bypass diverse protein obstacles (Fig. 4), and to recognize a lesion on a nucleosome-coated DNA (Fig. 6b). In contrast, sliding of *Escherichia coli* MutS on DNA is blocked by *Eco*RI(E111Q)^16^ and sliding of human and yeast Msh2–Msh6 is inhibited by nucleosomes^17^,^18^ (also see Supplementary Fig. 6).

How do Msh2–Msh3 and Msh2–Msh6 differ in their ability to bypass protein obstacles? We propose a model where the Msh2–Msh3 DNA-binding clamp transiently opens and closes as the protein slides on DNA (Fig. 6d). Msh2–Msh3 scans DNA via a combination of 1D sliding and hopping (steps 1 and 2 in Fig. 6d). Transient opening and re-closing of the DNA-binding clamp allows the protein to hop by briefly dissociating and re-engaging the DNA track (step 3 in Fig. 6d). Hopping facilitates long-range movement between two segments of DNA, as well as bypass of protein obstacles. Remarkably, Msh2–Msh3 is able to re-establish contact with the DNA such that it can recognize an extrahelical lesion after it hops over a nucleosome (step 4 in Fig. 6d). In contrast, we propose that Msh2–Msh6 and bacterial MutS have less dynamic DNA-binding clamps, which precludes hopping and obstacle bypass (Fig. 6d, right panel).

We reasoned that the different dynamic behaviours of Msh2–Msh3 and Msh2–Msh6 stem from the distinct types of DNA lesions that are recognized by each complex. Msh2–Msh6 recognizes mismatches and small insertion/deletion loops^14^,^15^. These lesions may lead to relatively moderate distortions of overall DNA duplex structure^59^,^60^,^61^. In contrast, Msh2–Msh3 chiefly recognizes some mismatches, large insertion/deletion loops and ssDNA flaps^14^,^15^. Such lesions are likely to lead to larger DNA distortions and are reflected in the differences between the Msh3 and Msh6 MBDs. The Msh3 MBD lacks a highly conserved phenylalanine-X-glutamate motif that directly interacts with DNA in bacterial MutS and Msh6 family proteins^43^,^44^. Instead, the Msh3 MBD encodes a conserved tyrosine–lysine pair that may result in looser contact with homoduplex DNA^34^,^43^,^44^,^52^. Similarly, the position of Msh2 is distinct in the context of the Msh3 MBD versus that of Msh6, making direct contacts with the lesion^52^. We tested our hypothesis by characterizing a chimeric Msh2–Msh6 that encodes the 130-residue MBD from Msh3. Remarkably, this chimeric construct gained the ability to hop on naked DNA and to bypass nucleosomes (Fig. 5).

Post-replicative MMR largely occurs during DNA replication, where ∼250 bp of newly replicated DNA is nucleosome-free for a short time^19^. Msh2–Msh6 is present at the replication fork and can thus scan this nucleosome-free region before the DNA is fully chromatinized^21^. Indeed, human Msh2–Msh6 is recruited to chromatin early in S-phase via the Msh6-encoded PWWP domain^62^. After DNA replication, iterative cycles of Msh2–Msh6 loading delays nucleosome deposition and may displace existing nucleosomes^63^,^64^,^65^,^66^, extending the time window for MMR on nucleosome-free DNA. Both Msh2–Msh6 and Msh2–Msh3 also function in HR, where these proteins block recombination between divergent DNA sequences (homeologous recombination)^21^,^26^. In homeologous recombination, Msh2–Msh6 recognizes mismatches within D-loops and clears RAD51 filaments, which are likely to be nucleosome-free^26^,^67^. In contrast, Msh2–Msh3 must locate ssDNA flaps that occur during HR and single-strand annealing. Both repair processes occur throughout the cell cycle and are not always coupled to DNA replication. In addition to potential direct recruitment via protein–protein interactions, Msh2–Msh3 may also need to recognize lesions via diffusion on a nucleosome-coated track. Further studies will be required to define how lesion binding and post-recognition complexes alter Msh2–Msh3 diffusion on a nucleosome-coated DNA.

In conclusion, our results demonstrate that Msh2–Msh3 diffuses on DNA via a combination of 1D sliding and hopping. Hopping is facilitated by transient opening of the DNA-binding clamp, which is in turn modulated by the Msh3 MBD. Msh2–Msh3 can hop over protein obstacles to recognize lesions that are on a nucleosome-coated DNA substrate. To our knowledge, this is the first direct demonstration that dynamic opening of a clamp-like DNA-binding domain can facilitate roadblock bypass during 1D-facilitated diffusion. These results provide insight into how a eukaryotic DNA repair factor bypasses roadblocks to function outside of replication-coupled MMR. More broadly, this study provides a paradigm for how fluctuations within DNA-binding domains may facilitate bypass of protein roadblocks during 1D-facilitated diffusion.

## Methods

### Buffers

Our typical imaging buffer conditions contained 40 mM Tris-HCl pH 8, 25–150 mM NaCl, 2 mM MgCl_2_, 2 mM DTT, 0.2 mg ml^−1^ BSA. The total ionic strength *I* was calculated using:


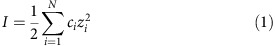


where *c* is the molar concentration of ion *i*, *z* is the charge number of that ion, and the sum is taken over all ions *N*. The pKa of Tris base is 8.1 at 25 °C, therefore 45% of the 40 mM Tris in our solution will be charged, contributing 9 mM to the total ionic strength. The hydrochloric acid used to titrate the Tris base down to a pH of 8.0 contributes 11 mM to the total ionic strength. Adding 2 mM MgCl_2_ contributes 6 mM to the total ionic strength (for the divalent magnesium ions, *z*^2^=4). Addition of 25–150 mM NaCl adds an additional 25–150 mM of ionic strength. Thus, we varied the total ionic strength (*I*) from 51 to 176 mM.

### Purification of Msh2–3 and Msh2–6

*Saccharomyces cerevisiae* Msh2HA–Msh3 and Msh2HA–Msh6 were purified by sequential ion exchange chromatography and ssDNA-affinity chromatography^27^,^34^. Msh2HA–Msh6 bound less tightly to Q-Sepharose fast flow (QFF, GE Life Sciences) than to PBE94 and therefore the starting NaCl concentration for the QFF column was reduced to 250 mM. Msh2HA–Msh6 eluted from QFF at ∼300 mM NaCl. *E. coli*-expressed Msh2HA–Msh6 and Msh2HA–Msh6(3MBD) were purified over QFF, ssDNA cellulose and QFF columns in the same manner as *S. cerevisiae*-expressed Msh2HA–Msh6 (refs 20, 68). Triple-FLAG epitope-tagged *Eco*RI(E111Q) was purified from *E. coli* using an intein–chitin-binding domain fusion construct (NEB IMPACT Kit)^30^.

### Purification of wild-type and 3 × FLAG hH2A

The wild-type or 3 × FLAG H2A plasmid was transformed into BL21(DE3) codon plus RIL cells (Agilent). A colony was inoculated into 50 ml LB broth with 50 μg ml^−1^ carbenicillin and 34 μg ml^−1^ chloramphenicol, and grown at 37 °C overnight. Fifteen millilitres of the overnight culture was seeded into 1.5 l LB broth and grown in the presence of both antibiotics. When the culture reached an OD_600_ of 0.6, 0.2 mM IPTG was added and the induction continued at 37 °C for 3.5 h. Cells were harvested at 5,000*g* for 15 min, and resuspended in 150 ml lysis buffer (100 mM NaPO_4_ pH 8.0, 8 M urea, 10 mM DTT, 15 mg benzamidine). Urea was deionized (501-X8 resin, Bio-Rad) immediately before use. Cells were lysed by sonication on ice, and centrifuged at 12 °C and 100,000*g* for 30 min. A 100 ml column was packed with 25 ml of SP-Sepharose Fast Flow resin (GE Healthcare), washed with 10 column volumes (CV) of water, and equilibrated with 10 CV of wash buffer (100 mM NaPO_4_ pH 8.0, 7 M urea, 10 mM DTT, 0.3 mM benzamidine). The 150 ml supernatant was added to the column and rotated for 1 h at room temperature (RT). The supernatant was washed with 5 CV of wash buffer, and eluted with five fractions of 5 ml elution buffer (100 mM NaPO_4_, pH 8.0, 1 M NaCl, 7 M urea, 10 mM DTT, 4 mM benzamidine). The resulting 25 ml eluent was loaded onto a Superdex-200 column (GE Healthcare) equilibrated in SAU-100 buffer (20 mM NaAcetate pH 5.2, 7 M urea, 100 mM NaCl, 1 mM EDTA, 5 mM β-mercaptoethanol). Gel filtration was performed using 120 ml SAU-100 buffer and the histone-containing fractions were loaded onto a tandem Q/SP column (10 ml each). After loading the histones, the tandem column was washed with 5 CV of SAU-100. The Q column was removed and 3 × FLAG H2A was eluted with a gradient from 0 to 100% SAU-600 (20 mM NaAcetate pH 5.2, 7 M urea, 600 mM NaCl, 1 mM EDTA, 5 mM β-mercaptoethanol) over 20 CV. The eluate was fractionated in 1.2 ml fractions and the histone-containing fractions were confirmed by SDS–PAGE. Protein concentration was determined by running an SDS–PAGE gel with BSA standards (Pierce Biotechnologies). Purified protein was lyophilized and stored in −80 °C. Both wild-type and 3 × FLAG H2A proteins purified with similar elution profiles and final yields.

### Inclusion body purification of histones

Each of the three histones (H2B, H3 and H4) was purified from inclusion bodies as previously described, with minor modifications^69^. Briefly, each histone was overexpressed in BL21(DE3) codon plus RIL cells. Cells were grown at 37 °C and 0.2 mM IPTG was added at OD_600_=0.6, followed by additional 3 h of induction at 37 °C. Cells were harvested by centrifugation at 5,000*g* for 20 min at RT. Cell pellets were suspended in 25 ml TW buffer (50 mM Tris-HCl pH 7.5, 100 mM NaCl, 1 mM EDTA), and stored at −80 °C until use. Each pellet was thawed and diluted up to 35 ml total volume using TW2 buffer (50 mM Tris-HCl pH 7.5, 100 mM NaCl, 1 mM EDTA, 5 mM β-mercaptoethanol, 1 mM benzamidine, and 1% (w/v) Triton X-100). Cells were lysed by sonication on ice for 2 min (10 s on—50 s off). To harvest the inclusion bodies, the lysate was centrifuged at 20,000*g* for 20 min at 4 °C. The pellets were rinsed with TW2 buffer by suspending, and centrifuged at 20,000*g* for 20 min at 4 °C. The pellet was washed in same way twice using TW2 without Triton X-100, and the final pellet was stored at −80 °C.

### Purification of histones

Each inclusion body pellet was mixed with 200 μl DMSO and 6.5 ml unfolding buffer (20 mM Tris-HCl, pH 7.5, 7 M guanidinium-HCl, and 10 mM DTT) by gently agitating for 1 h at RT, and centrifuged at 20,000*g* for 20 min at 4 °C. This was repeated two more times, and the supernatant from each centrifugation was dialyzed against 1 l urea buffer (10 mM Tris-HCl pH 8.0, 7 M urea, 1 mM EDTA, 5 mM β-mercaptoethanol, and 100 mM NaCl for H2B, 200 mM NaCl for H3 and H4) using 3,500 or 7,000 MWCO dialysis tubing (SnakeSkin, Pierce Biotechnologies). A tandem Q/SP column was equilibrated with 10% buffer B (10 mM Tris-HCl pH 8.0, 7 M urea, 1 mM EDTA, 1 mM DTT, 1 M NaCl) and 90% buffer A without 1 M NaCl. The dialyzed histones were loaded and washed with 10% buffer B. H2B was eluted from 10 to 40% buffer B over 20 CV in 200 min and H3 and H4 were eluted from 20 to 50% buffer B over 20 CV. The purified histones were checked by SDS–PAGE, lyophilized and stored at −20 °C until use.

### DNA substrates for total internal reflection fluorescence microscopy

DNA substrates for single-molecule experiments were prepared by annealing oligonucleotides IF003 and IF004 to λ-phage DNA (Supplementary Table 4; New England Biolabs). Briefly, ∼15 nM λ-phage DNA was heated to 65 °C, combined with 1 μM IF003 and IF004, and allowed to slowly cool to RT. After cooling, the reaction was supplemented with ATP to 1 mM, T4 DNA ligase (2,000 units; New England Biolabs) and incubated overnight at RT. The ligase was heat inactivated and the reaction was passed over an S-1000 gel filtration column (GE) to remove excess proteins and oligonucleotides. The DNA was stored at 4 °C or immediately isopropanol precipitated for nucleosome reconstitution.

### Histone octamer assembly

Each of the four histone was dissolved in unfolding buffer (20 mM Tris-HCl pH 7.5, 7 M guanidinium-HCl, and 10 mM DTT), and gently agitated for 1 h at RT. The histones were mixed in equimolar ratios of H3/H4, and a 10% higher molar ratio of H2A/H2B relative to H3/H4. The mixture was adjusted to a final concentration of 1 mg ml^−1^ and dialyzed against refolding buffer (10 mM Tris-HCl pH 8.0, 1 mM EDTA, 5 mM β-mercaptoethanol, 2 M NaCl) using 3,500 MWCO dialysis tubing with several buffer exchanges over 48 h. The dialyzed mixture was centrifuged to remove aggregates, and concentrated using spin-concentrators (Amicon Ultra-15; Millipore) to a final volume of about 1 ml. Gel filtration over a Superdex-200 (GE Healthcare) using SAU-200 was performed to resolve histone octamers from dimers and tetramers in the refolding buffer. The octamer peak fractions were combined, concentrated using a 10,000 MWCO spin-concentrator (Amicon Ultra-4, Millipore), and flash frozen using liquid N_2_. The resulting histone octamers were stored in −80 °C until use.

### Nucleosome reconstitution

To reconstitute human nucleosomes on the λ-phage DNA substrate, the DNA was first ligated to biotinylated and DIG-terminated oligonucleotides (IF003 and IF004, respectively) and gel-filtered through an S-1000 column (GE). The DNA was concentrated using isopropanol precipitation, and dissolved to a final concentration of 70 ng μl^−1^ in TE with high salt (10 mM Tris-HCl pH 8.0, 1 mM EDTA, 2 M NaCl). For reconstitution, 30 μl of the DNA (final concentration of ∼20 ng^−1^ was used in total volume of 100 μl. The octamer was diluted 10-fold in dilution buffer (10 mM Tris-HCl pH 7.6, 1 mM EDTA, 2 M NaCl) right before use. The 100 μl mixture was dialyzed using a mini dialysis button (10 K MWCO, Bio-Rad) against 400 ml storage buffer (10 mM Tris-HCl pH 7.6, 1 mM EDTA, 1 mM DTT) that contained gradually decreasing concentrations of NaCl. Dialysis was performed in a cold room at 4 °C for at least 90 min for each 1.5 M, 1 M, 0.8 M, 0.6 M, 0.4 M NaCl containing storage buffer. 0.2 M NaCl buffer was used for overnight dialysis. At a nominal input ratio of 1:75 (DNA:octamer), we counted approximately 1–5 nucleosomes per DNA molecules. The large nominal DNA:octamer ratio probably stems from octamer loss due to aggregation onto the dialysis membrane and polypropylene tubing during the extended dialysis procedure^70^. The nucleosome-coated DNA was stored at 4 °C for up to two weeks.

### Msh2–Msh3 DNA-binding and ATPase activities

DNA substrates for the gel mobility shift and ATPase assay were radio-labelled (where appropriate) using homoduplex (LS1/LS2), +1 loop (LS2/LS6T), +8 loop (LS2/LS8) and 3′-ssDNA flap (LS1/LS3/LS16) (Supplementary Table 4)^34^. Next, we performed titrations of Msh2HA–Msh6, Msh2HA–Msh3 and Msh2HA–Msh6(3MBD) according to standard protocols^34^,^35^. Briefly, each protein was incubated at the indicated concentration with 1 nM labelled DNA substrate, 20 mM HEPES (pH 7.5), 100 mM NaCl. 1 mM DTT, 40 μg ml^−1^ BSA, 2 mM MgCl_2_ in a total of 10 μl. The reactions were assembled on ice and then incubated at RT for 5 min following the addition of the DNA substrate. The reactions were electrophoresed through a 4%, ½ X TBE polyacrylamide gel at 130 V for 45 min in the cold. The gels were dried, exposed to a PhosphorImager screen (Molecular Dynamics) and quantified with ImageQuant (GE Life Sciences).

For gel mobility shift assays in the presence of antibody and/or QDs, Msh2HA–Msh3 (200 (+) or 400 (++) nM) was incubated with stoichiometric concentrations of αHA antibody, αFLAG antibody or αHA-coupled QDs on ice for 15 min before the addition of the labelled DNA substrate. In these reactions, 20 nM DNA substrate was included. The reactions were then incubated at RT for 10 min and electrophoresed through a 3%, ½ X TBE gel at 145 V for 90 min. The gels were dried, exposed to a PhosphorImager screen (Molecular Dynamics) and quantified with ImageQuant (GE Life Sciences).

The ATPase assays were performed in 5 μl reactions at 100 nM protein complex, 25 mM Tris (pH 7.5), 2 mM MgCl_2_, 1 mM DTT and 40 μg ml^−1^ BSA. When present, αHA-QDs were incubated with the protein on ice at stoichiometric concentrations for 15 min before addition to the reaction. DNA template was added to 2 μM final concentration and was incubated with protein for 5 min at RT. ATP spiked with γ^32^P-ATP was added last at increasing concentrations (0, 20, 33, 50, 67, 100 and 250 μM ATP). The reaction was incubated at 30 °C for 30 min and then quenched by the addition of EDTA. The fraction hydrolyzed was determined by PEI-cellulose TLC with 0.6 M potassium phosphate buffer pH 3.4. The data were analysed in Prism (GraphPad).

### Single-molecule microscopy

Images were collected with a Nikon Ti-E microscope in a prism-TIRF configuration. The inverted microscope setup allowed for the sample to be illuminated by a 488 nm laser light (Coherent) through a quartz prism (Tower Optical, Co.). To minimize spatial drift, experiments were conducted on a floating TMC optical table. A 60 × water immersion objective lens (1.2 NA, Nikon), two EMCCD cameras (Andor iXon DU897, −80 °C) and NIS-Elements software (Nikon) were used to collect the data with a 200 ms exposure time. Two-color imaging was conducted using a 638 nm dichroic beam splitter (Chroma). Frames were saved as TIFF files without compression for further image analysis in ImageJ (NIH). All single-molecule results were the product of at least two independent experiments.

### Quantum dots

QDs were conjugated to Rabbit anti-HA tag antibodies (ICL labs #RHGT-45A-Z) or Mouse anti-FLAG tag antibodies (Sigma #F3165) using SiteClick antibody labelling kits (Life Technologies) according to the manufacturer's instruction. The unconjugated antibodies were removed using a HiPrep Sephacryl S-300 HR gel filtration column (GE). QDs were stored in PBS (137 mM NaCl, 2.7 mM KCl, 9.1 mM K_2_HPO_4_ and 2.8 mM KH_2_PO_4_) at 4 °C.

### Fluorescent labelling of MMR complexes

The Msh2 subunit encodes an HA epitope tag between amino acids 644 and 645. HA epitope-tagged Msh2–Msh3, Msh2–Msh6(3MBD), and Msh2–Msh6 were labelled with anti-HA conjugated QDs^27^. Briefly QDs and protein were co-incubated at a 1:1 molar ratio (150 nM protein and QDs) in BSA buffer (40 mM Tris-HCl pH 8.0, 1 mM DTT, 2 mM MgCl_2_, 0.2 mg ml^−1^ BSA) for 15 min on ice followed by dilution to a final concentration of 5–10 nM in BSA buffer containing the experimentally indicated concentration of NaCl (25–150 mM) and nucleotide (1 mM ADP, ATP, or AMP–PNP; Roche). The diluted protein–QD mixture was injected into the flowcell, and allowed to incubate with DNA curtains for 5–10 min. After incubation, excess QDs and all non-DNA-bound proteins were flushed out of the flowcells, buffer flow was terminated, and data acquisition was initiated. For experiments where the protein lifetime on DNA was <30 s (Msh2–Msh3 and Msh2–Msh6(3MBD) at NaCl concentrations above 75 mM), free protein and QDs were retained in the flowcell. This allowed for repeated rounds of protein binding and rapid dissociation. The short lifetimes guaranteed that individual DNA molecules accumulated fewer than three protein complexes.

### Fluorescent labelling of *Eco*RI(E111Q) and nucleosomes

Triple FLAG epitope-tagged *Eco*RI(E111Q) was incubated with DNA at 0.5 nM concentration in buffer containing 10 mM Tris pH 7.8, 1 mM EDTA, and 150 mM NaCl. The reaction was then diluted 10-fold with BSA buffer and injected into the flowcell. 3 × FLAG-*Eco*RI(E111Q) was labelled *in situ* by injecting 10 nM of anti-FLAG conjugated QDs directly into the flowcell at a rate of 100 μl min^−1^. Before injection, anti-FLAG conjugated QDs were diluted to 5 nM in BSA buffer containing the experimentally indicated concentration of NaCl and nucleotide. Nucleosomes were reconstituted with 3 × FLAG H2A containing octamers. The nucleosomes were labelled *in situ* with a strategy identical to *Eco*RI(E111Q).

### Data analysis of particle tracking

Fluorescent particles were tracked in ImageJ with a custom-written particle tracking script (available on request). The resulting trajectories were analysed in Matlab (Mathworks). For each image frame the fluorescent particle was fit to a two-dimensional Gaussian function to obtain its position with sub-pixel resolution. The series of positions of a given particle were used to obtain trajectories. To ensure that trajectories corresponded to proteins on DNA, only molecules that responded to buffer flow controls and diffused for a minimum of 10 s were analysed. Only DNA-bound QDs were counted for statistical analysis.

### Data analysis of measuring diffusion coefficients

For all diffusion experiments, we used double-tethered DNA curtains, which extend DNA molecules between two nano-fabricated chromium features in the absence of buffer flow^28^. The average separation between the two chromium features was 13 μm (∼80% extension relative to B-form DNA). Trajectories of individual molecules were used to calculate the 1D MSD as a function of time interval using:


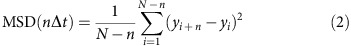


where *N* is the total number of frames in the trajectory, *n* is the number of frames for a given time interval, Δ*t* is the time between frames, and *y*_*i*_ is the position of Msh2–Msh3 at frame *i*. To minimize systematic errors associated with estimating diffusion coefficients for very short trajectories, we only considered particles that diffused for more than 10 s (50 frames). The MSD was calculated for the first 10 time intervals (Δ*t*=0.2 s-2 s) and plotted as a function of Δ*t* to generate the line:


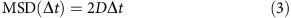


where *D* is the diffusion coefficient. Plots were fit to a line and the fit was used to calculate diffusion coefficients of individual molecules. Diffusion coefficients were calculated for ⩾45 molecules per condition, and are reported as a mean±s.d. Statistical analysis was performed on the Logarithm of the data using a two tailed student's *t*-test, with a 95% confidence interval.

### Characterizing DNA transfer events

We scored the frequency of protein transfer between two DNA molecules that were tethered to adjacent pedestals (separated by 1 μm). Msh2–Msh3, Msh2–Msh6(3MBD) or Msh2–Msh6 was injected into the flowcell and allowed to diffuse on DNA for at least 10 min. After the diffusion traces were collected, the DNA molecules were visualized via fluorescent staining. Our criteria for identifying Msh-protein transfer between two adjacent DNA molecules were: (i) the protein first diffused in register with one DNA for greater than 4 s, (ii) the protein shifted positions to the neighbouring DNA in less than one frame (200 ms) and (iii) the same protein continued to diffuse in register with an adjacent DNA for at least 4 additional seconds. All DNA transfer experiments were performed in imaging buffer with 1 mM ADP and 100 mM NaCl.

### Characterizing roadblock bypass events

Roadblock bypass was observed using the following DNA roadblocks on double-tethered DNA: Msh2–Msh3, *Eco*RI(E111Q) and nucleosomes. For Msh2–Msh3 roadblocks, Msh2–Msh3 was divided into two 100 nM reactions individually labelled with QDs that fluorescently emit at either 605 or 705 nm. The reactions were incubated for 15 min on ice and diluted 10-fold in BSA buffer supplemented with 10 mM NaCl and 1 mM ADP. The reactions were then mixed and injected into the flowcell. After DNA incubation, the buffer was switched from 10 to 100 mM NaCl using a six port, two position manual valve (Valco Instruments, Co., Inc.). After buffer exchange, the proteins were visualized on DNA for ∼10 min

For *Eco*RI(E111Q), the hydrolytically dead restriction enzyme was first incubated with DNA and labelled with QDs *in situ*^30^. Next, 10 nM of QD-labelled Msh2–Msh3 was injected into the flowcell. Buffer exchange and imaging was done under identical conditions as for Msh2–Msh3 on naked DNA. Nucleosome bypass experiments were also done using the same buffer exchange strategy. We checked whether the Msh2–Msh3 nucleotide state altered nucleosome bypass probabilities by repeating these experiments with 1 mM ADP, AMP–PNP or ATP–Mg^+2^, respectively. Nucleosomes were labelled *in situ* as described for *Eco*RI(E111Q). To test the effect that QDs had on roadblock bypass, nucleosomes were labelled either before Msh2–Msh3 injection or after Msh2–Msh3 image acquisition.

We considered a protein to pass a roadblock if it satisfied the following criteria: (a) the protein was initially diffusing on one side of the roadblock, (b) the protein transiently co-localized with the roadblock within a ‘collision zone' of 750 bp (200 nm) and (c) the diffusing protein clearly continued diffusing on the opposite side of the roadblock. The collision zone is defined as 3 × the s.d. of the random motion exhibited by a stationary protein on double-tethered DNA curtains over >100 frames. This random motion is the result of thermal fluctuations experienced by the DNA.

### Lysogen DNA purification

The *E. coli* lysogen used to generate flap-containing λ-DNA was a generous gift from the Greene lab. The lysogen was created by replacing the region between the *Ngo*MIV and the *Xba*I restriction enzyme cutsites (20,041 bp and 24,374 bp, respectively) with a 151 bp DNA segment containing three *Bsp*QI sites and a unique *Nco*I cut site. To purify the DNA, a single *E. coli* colony was grown to confluency in 50 ml of LB broth overnight at 30 °C and used to inoculate 500 ml of LB broth. When OD_600_=0.6, the flasks were rapidly heated to 42 °C followed by a 15 min heat shock at 45 °C. The cells were then grown at 37 °C for 2 h. Cells were pelleted at 3,000*g* for 30 min, and suspended in SM buffer (50 mM Tris-Cl pH 7.5, 100 mM NaCl and 8 mM MgSO_4_). Cells were lysed with 2% chloroform, and the genomic nucleic acids degraded with 50 ng μl^−1^ of DNAseI and 30 ng μl^−1^ RNAse A (Sigma). The lysate was clarified by centrifugation at 6,000*g* for 15 min. Phage heads were precipitated using cold buffer L2 (30% PEG 6000, 3 M NaCl) for 30 min at 4 °C. Phage heads were pelleted by centrifuging at 10,000*g* for 10 min. The phage pellet was suspended in buffer L3 (100 mM Tris pH 7.5, 100 mM NaCl and 25 mM EDTA). Phage heads were degraded by buffer L4 (4% SDS) and 100 μg ml^−1^ of Proteinase K (NEB). SDS was precipitated by adding buffer L5 (3 M potassium acetate pH 5.5) followed by centrifugation at 15,000 g for 30 min. The supernatant was passed over a pre-equilibrated QIAGEN Genomic-tip 500 DNA purification column (QIAGEN). DNA was purified from the columns according to the manufacturer's instruction, and dissolved in Te buffer (10 mM Tris pH 7.5, 0.1 mM EDTA). DNA was stored in 4 °C for up to 6 months.

### Flap incorporation

50 μg of purified λ-DNA (see above) was diluted in NEB buffer 3 and digested with 50,000 units of Nt.BspQI (NEB) for 1 h at 55 °C. Oligonucleotides IF003 and IF004, along with oligonucleotides incorporating either a 3′-ssDNA flap (MB34, MB35), a digylated 3′-ssDNA flap (MB35,MB36) or a complementary sequence (MB32) were added in 500 × molar excess to the nicked λ-DNA (Supplementary Table 4). The reaction was heated to 70 °C and cooled at a rate of 0.5 °C min^−1^ to promote oligonucleotide incorporation. The reaction was supplemented with 1 mM of ATP and 3,000 units of T4 DNA ligase (NEB) and incubated overnight at RT. Reactions were quenched with 50 mM EDTA. An aliquot of the reaction was taken for agarose gel insertion diagnostics, while the remainder was incubated with five units of Proteinase K (NEB) at 50° for 30 min to degrade Nt.BspQI and T4 DNA ligase. The DNA was passed over an S-1000 gel filtration column (GE) and stored at 4 °C.

### Characterizing flap insertion efficiency

Insertion of the flap oligonucleotide abolishes an *Nco*I site within the λ-DNA. *Nco*I cleavage can thus be used to monitor the oligo insertion efficiency. After the flap oligonucleotide was ligated into the nicked λ-DNA, a Bio-Spin size exclusion column (Bio-Rad) was used to exchange the buffer into NEB Buffer 4. 1.5 μg of the λ-DNA was digested with 20 units of *Nco*I-HF (NEB) at 37 °C for 1 h. Digests were run on a 0.8% agarose gel containing 0.5 μg ml^−1^ of ethidium bromide (Apex) for three hours at 100 V. Gels were imaged with a Typhoon gel imager (GE).

Alkaline agarose gels were used to monitor complete re-ligation of the nicked λ-DNA. 1.5 μg of proteinase K treated DNA was loaded into a 0.6% alkaline agarose gel using 6 × alkaline gel-loading buffer (300 mM NaOH, 6 mM EDTA, 18% (w/v) glycerol and 0.15% (w/v) Orange G (NEB)). Gels were run in 1x alkaline electrophoresis buffer (50 mM EDTA pH 8.0, 1 M NaOH) for 24 h at 20 V and 4 °C. Gels were incubated in neutralization buffer (1 M Tris-Cl pH 7.6, 1.5 M NaCl) for 45 min at RT, stained in a solution of 1 × TAE and 0.5 μg ml^−1^ ethidium bromide for 30 min, and de-stained by soaking in ddH_2_0 for 20 min. Gels were imaged with a Typhoon gel imager.

### Visualizing via single-molecule fluorescence imaging

To directly visualize the flap oligonucleotide within DNA curtains, oligonucleotides MB35 and MB36 were inserted into the DNA to generate a substrate with a 3′-ssDNA flap terminated with a DIG label. The oligonucleotide was incorporated into the DNA substrate, purified and assembled into DNA curtains. The digylated DNA was labelled *in situ* with 12.5 ng μl^−1^ of DIG monoclonal antibody (Life Technologies #700772) followed by 10 nM of Goat anti-Rabbit QD605 conjugate (Life Technologies #Q-11401MP). The average signal from 150 frames was used to calculate the QD position.

## Additional information

**How to cite this article:** Brown, M. W. *et al*. Dynamic DNA binding licences a repair factor to bypass roadblocks in search of DNA lesions. *Nat. Commun.* 7:10607 doi: 10.1038/ncomms10607 (2016).

## Supplementary Material

Supplementary Information Supplementary Figures 1-8, Supplementary Tables 1-4 and Supplementary References
