# Supplementary material for: Dynamic DNA binding licenses a repair factor to bypass roadblocks in search of DNA lesions
Source: Nat Commun. 2016 Feb 3;7:10607. doi: 10.1038/ncomms10607 (PMC4742970; doi:10.1038/ncomms10607)
Supplement: Supplementary Information — Supplementary Figures 1-8, Supplementary Tables 1-4 and Supplementary References [file ncomms10607-s1.pdf]

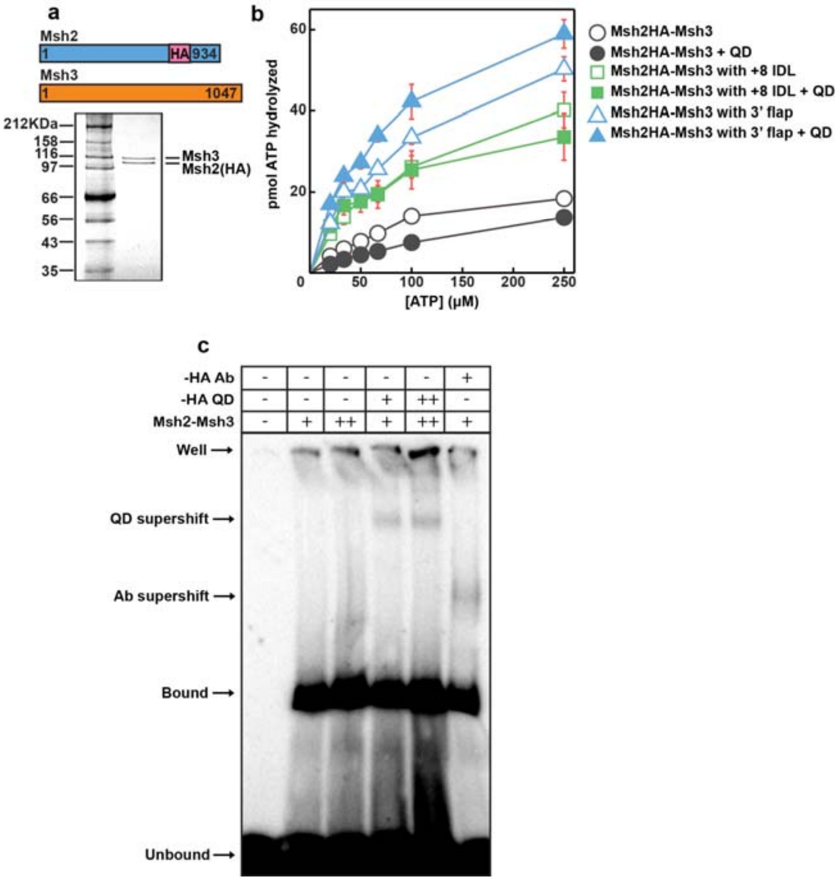

Supplementary Figure 1. Biochemical characterization of yeast Msh2-Msh3. (a) An SDS-PAGE gel of purified Msh2-Msh3. **(b)** ATPase assays indicates that QD-conjugated Msh2-Msh3 retains near-wild type ATPase activity in the absence of DNA (black), on a +8 insertion/deletion loop (IDL, green), and with a 3'-ssDNA flap (blue). Empty symbols: no QDs, filled symbols: conjugated to anti-HA QDs. Error bars are standard deviation of at least three measurements. **(c)** Electrophoretic mobility shift assay (EMSA) for Msh2-Msh3 on a 3' ssDNA flap DNA substrate. QD- and antibody-conjugated and wild type proteins retain affinity for 3'-ssDNA flap DNA. The DNA substrate was made by annealing oligos LS1/LS3/LS16 (Supplementary Table 4). Additional details are included in the supplementary methods.

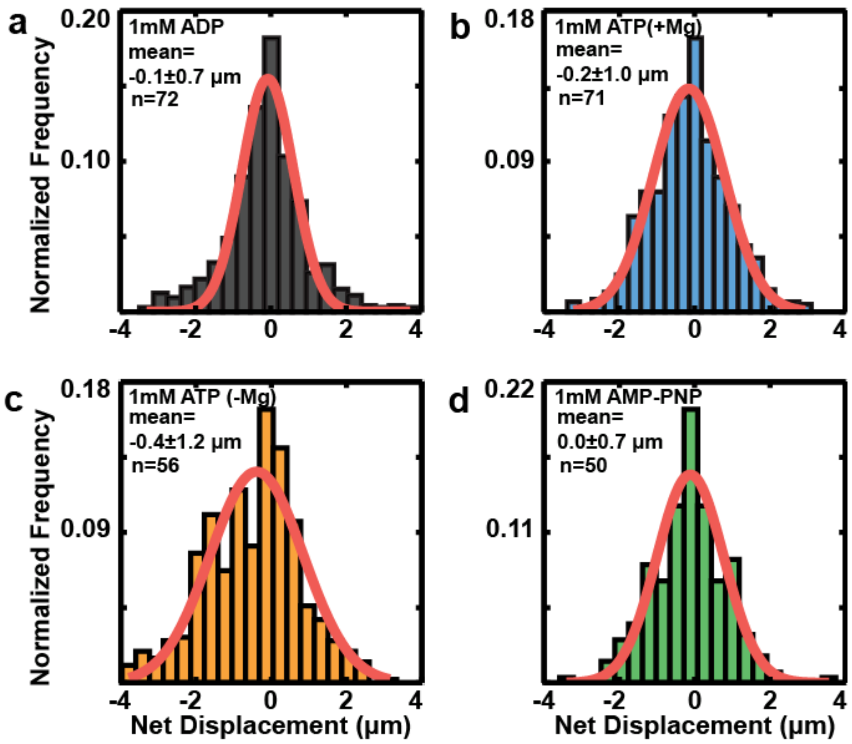

Supplementary Figure 2. Quantitative analysis of Msh2-Msh3 diffusion. Panels **(a)-(d)** summarize the net displacement of at least 50 Msh2-Msh3 molecules at the indicated nucleotide concentration. The red line is a Gaussian fit through the data. The mean ± S.D. are indicated in each panel. Zero net displacement is a signature of thermally driven 1D-diffusive motion.

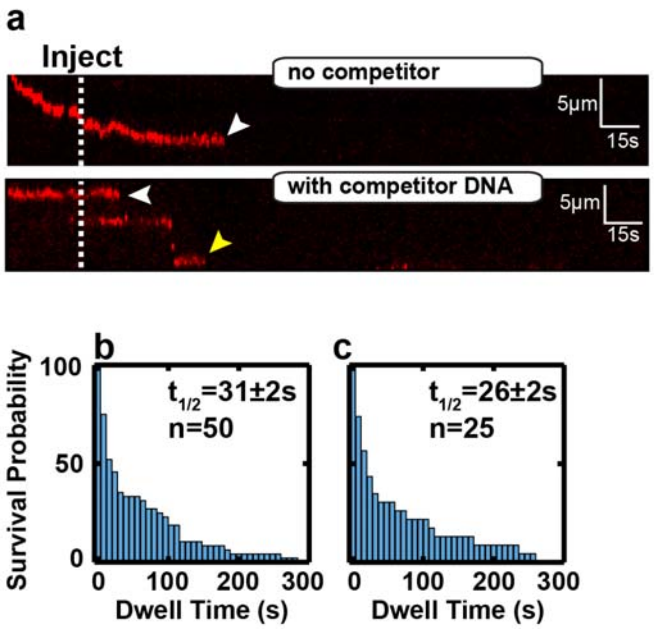

Supplementary Figure 3. Msh2-Msh6 dwell times are not sensitive to the addition of competitor DNA. (a) Kymograph of Msh2-Msh6 on single-tethered DNA curtains (top) and in the presence of the same competitor DNA as in Figure 3a (bottom). White arrowheads represent dissociation from the middle of the DNA and the yellow arrowhead shows dissociation from the free DNA end. The dashed vertical line defines when the competitor DNA (or a mock injection) was introduced into the flowcell. **(b)** Quantification of the Msh2-Msh6 survival probabilities with no competitor or **(c)** homoduplex competitor DNA. The half-life ± S.E. is included in each panel.

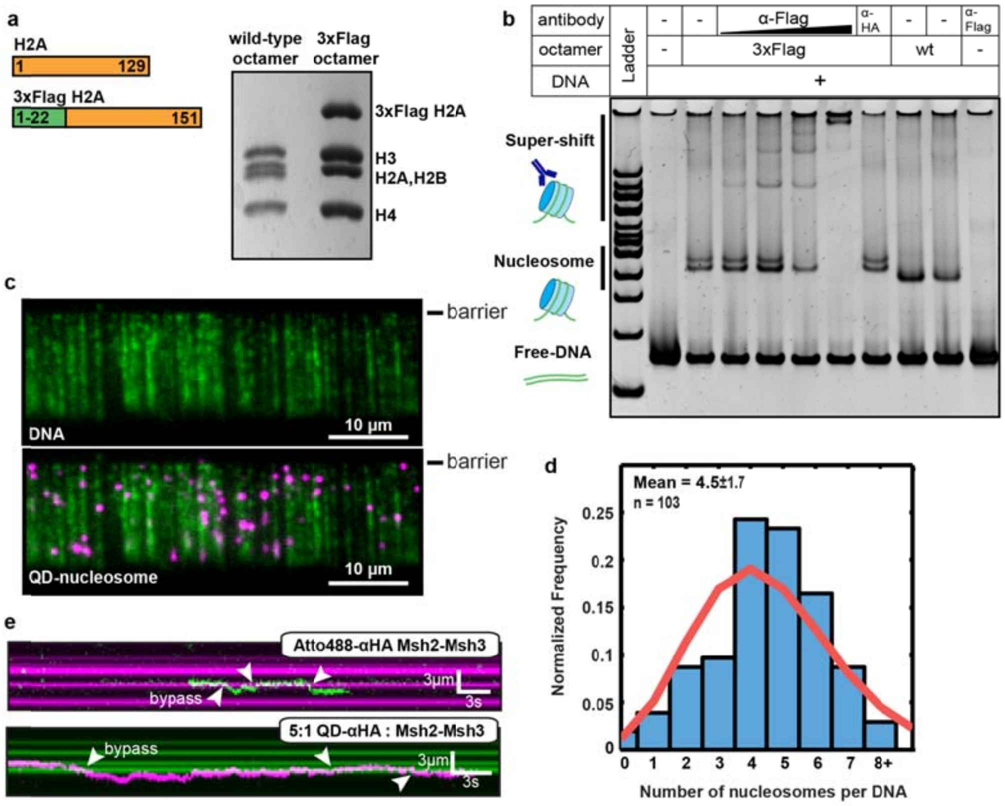

Supplementary Figure 4. Characterization of reconstituted nucleosomes. (a) SDS-PAGE of unlabeled (wild type) and epitope tagged recombinant histone octamers. For fluorescent labeling, a 3xFlag epitope tag was added at the N-terminus of H2A. **(b)** Reconstitution of epitope-tagged and wild type histone octamers on a high nucleosome affinity (Widom "601") DNA substrate[1]. Epitope-tagged—but not wild type—nucleosomes could be further supershifted by an anti-Flag antibody. Anti-HA (αHA) antibodies did not interact with Flag-tagged nucleosomes. **(c)** DNA curtain (top) with fluorescent nucleosomes that were deposited via a salt dialysis procedure. The DNA (green) is stained with YOYO-1 and the nucleosomes are labeled with anti-FLAG conjugated QDs (705 nm emission). The DNA is not degraded by the nucleosome reconstitution. **(d)** Quantification of the number of nucleosomes per DNA molecule. A Gaussian fit through the data (red) indicates 4.5±1.7 (denotes S.D.; n=103) nucleosomes per DNA. (**e**) Top panel: kymograph of an Atto488-αHA labeled Msh2-Msh3 (green) hopping over a nucleosome (magenta). Hopping over nucleosomes was observed in 43% (n=17/40) of the single-molecule trajectories, as observed with QD-labeled Msh2-Msh3. Bottom panel: Msh2-Msh3 can also hop over obstacles when it is pre-labeled with a large excess of αHA-QDs. We estimate substantially fewer than one protein per QD at these conjugation ratios[2]. These results highlight that Msh2-Msh3 hopping is independent of the QD label.

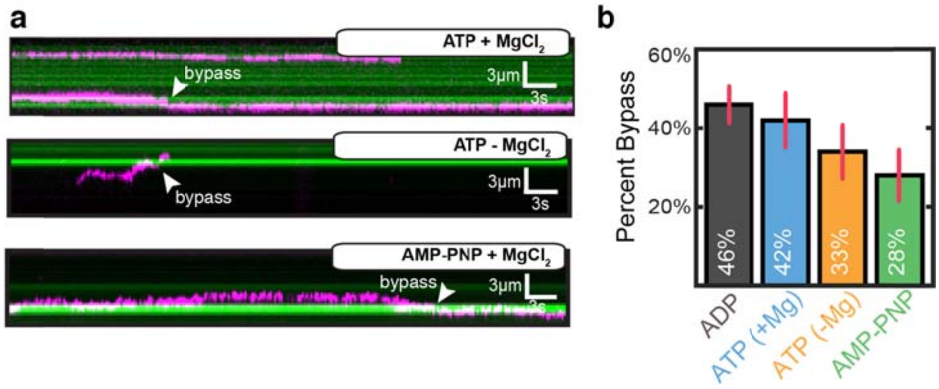

Supplementary Figure 5. Nucleotides modulate Msh2-Msh3 bypass of nucleosome obstacles. (a) Kymograph of Msh2-Msh3 (magenta) bypassing nucleosomes (green) in buffers containing 1 mM of the indicated nucleotide. Nucleosomes were post-labeled with α-FLAG QDs and the collisions are indicated with white arrowheads. **(b)** Quantification of the bypass frequency with 1 mM of the indicated nucleotide. N>50 for all nucleotide conditions. Error bars represent the S.D. obtained via bootstrapping analysis.

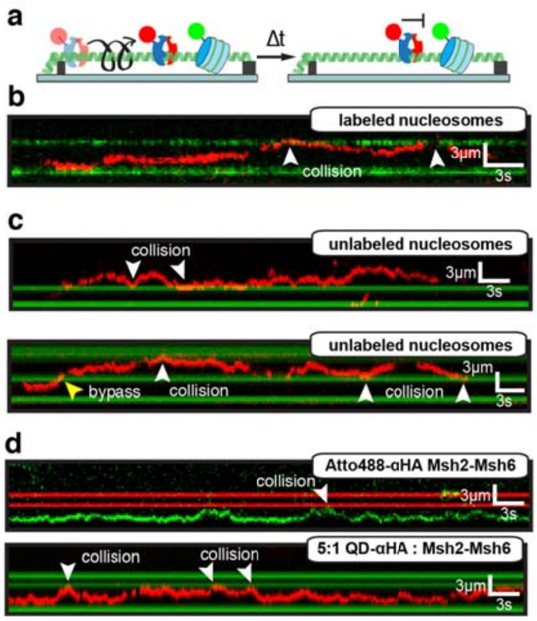

Supplementary Figure 6. Msh2-Msh6 rarely bypasses nucleosome obstacles. (a) Cartoon illustration of Msh2-Msh6 colliding with pre-labeled fluorescent nucleosomes. **(b)** Kymograph of a sample Msh2-Msh6 nucleosome trajectory. Collisions are indicated as white arrowheads. We never saw Msh2-Msh6 bypassing QD-labeled nucleosomes (n=31 trajectories). **(c)** Kymographs of collisions between Msh2-Msh6 and post-labeled nucleosomes. Typically, Msh2-Msh6 collides with a nucleosome, but does not hop over the roadblock (91% or 91/100 events, top panel). Occasionally (9% or 9/100 of Msh2-Msh6 trajectories), we saw Msh2-Msh6 bypassing an unlabeled nucleosome (yellow arrowhead). These results are quantified in Figure 5e. **(d)** Top panel: kymograph of an Atto488-αHA labeled Msh2-Msh6 (green) blocked by a nucleosome (magenta). 87% of the Atto488-αHA labeled Msh2-Msh6 complexes were blocked by unlabeled nucleosomes (n=35/40). Bottom panel: Msh2-Msh6 is also blocked by nucleosomes when it is pre-labeled with a large excess of αHA-QDs (red: Msh2-Msh6, green: nucleosomes).

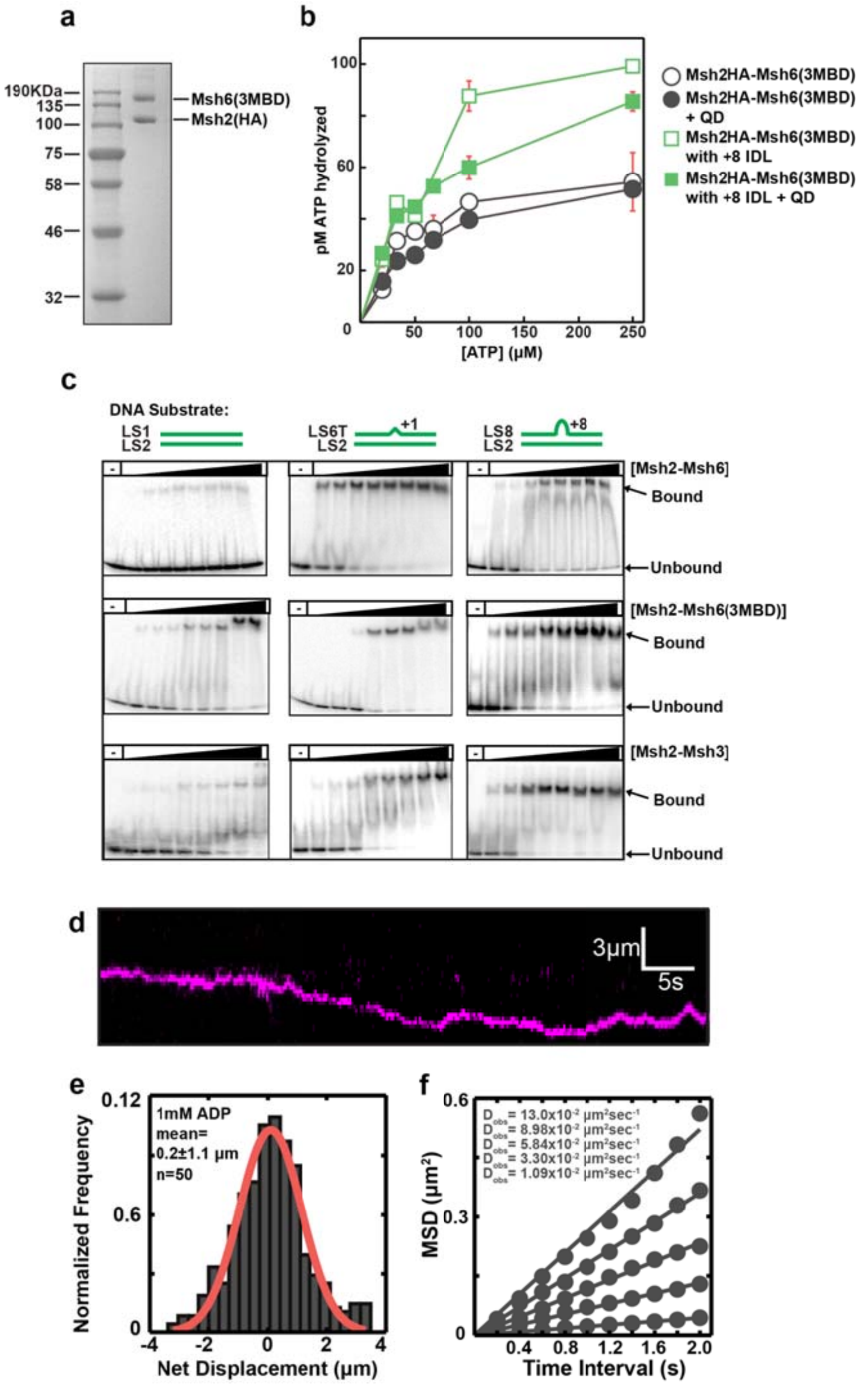

Supplementary Figure 7. Characterization of Msh2-Msh6(3MBD). (a) The protein was deemed to be >95% pure as judged by SDS-PAGE. **(b)** ATPase activity of the wild type and fluorescently labeled Msh2-Msh6(3MBD) in the absence of DNA (black), or on a +8 insertion/deletion loop (IDL, green). Empty symbols: no QDs, filled symbols: conjugated to anti-HA QDs. Error bars are standard deviation of at least three

measurements. **(c)** EMSA characterization of Msh2-Msh6, Msh2-Msh6(3MBD), and Msh2-Msh3 lesion affinity. In all assays, the DNA concentration was 1 nM. In lanes 2-9, the protein concentration was: 10 nM, 20 nM, 40 nM, 80 nM, 100 nM, 150 nM, 200nM, and 300nM, respectively. The lesion specificity of Msh2-Msh6(3MBD) is between those reported for Msh2-Msh6 and Msh2-Msh3. These findings are consistent with previous SPR results described by Dowen et. al. (ref. [3]) **(d)** A kymograph of fluorescent Msh2-Msh6(3MBD) diffusing on homoduplex DNA. **(e)** Net displacement of 50 Msh2-Msh6(3MBD) trajectories. The red line is a Gaussian fit to the data with a mean of 0.2±1.1 μm (S.D.). **(f)** A linear fit through the mean squared displacements for five representative trajectories. The slopes of the fits were used to obtain 1D diffusion coefficients.

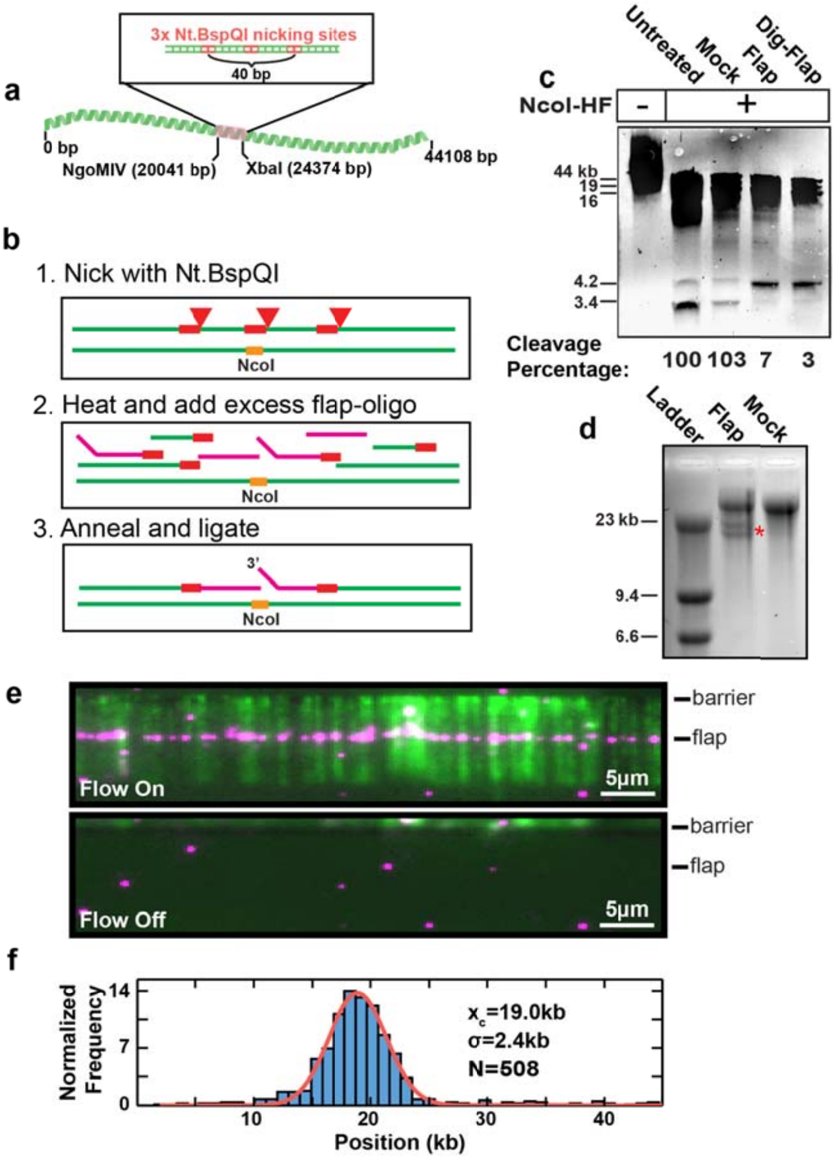

Supplementary Figure 8. DNA substrates with an extrahelical lesion. (a) A nicking cassette with three Nt.BspQI sites was cloned 20kb upstream of the biotinylated DNA end (between the NgoMIV and XbaI restriction sites). The three nicking sites are each separated by 20 nucleotides. The DNA is purified from phage lysogens as described in the Supplementary Methods. **(b)** The strategy for inserting an ssDNA flap into the DNA substrate. The DNA is nicked with Nt.BspQI, heated to 71˚C in the presence of 1000-fold molar excess of flap oligonucleotides, cooled slowly, and incubated overnight with T4 ligase. The 3' ssDNA-flap disrupts an existing NcoI restriction site. **(c)** Cleavage with NcoI is used to quantify the degree of flap incorporation. Cutting untreated (lane 2) or mock-treated (lane 3) DNA with NcoI liberates two well-resolved bands (4.2 and 3.4 kb). Flap insertion abolishes one NcoI site, leading to the disappearance of the 3.4 kb band

(lanes 4 & 5). Cleavage efficiency is defined as the intensity of the 3.4 kb band divided by the sum of intensities of the 3.4 and 4.2 kb bands. Cleavage efficiency was normalized to untreated λ-DNA (lane 2). **(d)** Alkaline agarose gel (0.6%) of the substrates shown in (c) shows efficient ligation of all 13 Nt.BspQI sites in our DNA substrate. As expected, the flap-containing DNA strand resolves into 44 kb, 24 kb and 20kb bands (red asterisk). When the reaction is carried out with a complementary homoduplex oligo (lane 3), we only see a 44 kb band (both top and bottom strands). **(e)** We confirmed that the flap was incorporated into the DNA by directly visualizing the flap-oligonucleotide in a single-tethered DNA curtain. For these experiments, the flap oligonucleotide was terminated with a 3'-digoxigenin (dig) and was labeled with anti-dig QDs (magenta). The DNA was stained with YOYO-1 (green). A line of QDs was visible at the expected flap position (top panel). Nearly all QDs retracted to the barrier when buffer flow was turned off (bottom panel). **(f)** Probability distribution of flap-QD positions. The red line is a Gaussian fit to the data (mean =19.0±2.4 kb; n=508; error values represent one standard deviation of the fit).

Supplementary Table 1. Nucleotide-dependent Msh2-Msh3 diffusion coefficients

	**1D Diffusion ± S.D. (μm^2 s^{-1})**	**Net Displacement ± S.D. (μm)**	**Number of Molecules**	**p-value (relative to ADP)**
**ADP**	(2.5±2.1) x 10^{-2}	-0.1±0.7	72	
**ATP**	(2.0±2.0) x 10^{-2}	-0.2±1.0	71	1.3 x 10^{-1}
**ATP-Mg^{+2}**	(3.8±3.9) x 10^{-2}	-0.4±1.2	56	2.5 x 10^{-2}
**AMP-PNP**	(5.3±5.8) x 10^{-2}	0.0±0.7	50	1.4 x 10^{-4}
**No nucleotide**	(3.7±3.5) x 10^{-2}	-0.1±0.9	55	1.2 x 10^{-2}

Note: All data points were acquired in imaging buffer with 50 mM NaCl (total ionic strength of 76 mM).

Supplementary Table 2. Sodium chloride-dependent Msh2-Msh3 diffusion coefficients

**[NaCl] (mM)**	**Total Ionic Strength (mM)**	**1D Diffusion ± S.D. (μm^2 s^{-1})**	**Net Displacement ± S.D. (μm)**	**Number of Molecules**	**p-value (relative to 25 mM NaCl)**
**25 mM**	51	(3.1±2.7) x 10^{-2}	-0.1±0.6	47	
**50 mM**	76	(2.5±2.1) x 10^{-2}	-0.1±0.7	72	1.1 x 10^{-1}
**75 mM**	101	(7.0±7.0) x 10^{-2}	0.1±1.0	60	6.7 x 10^{-5}
**100mM**	126	(1.0±0.8) x 10^{-1}	0.0±1.4	52	5.8 x 10^{-10}
**150mM**	176	(1.2±1.4) x 10^{-1}	-0.1±1.7	49	7.5 x 10^{-7}

Note: All data points were acquired in imaging buffer with 1 mM ADP.

Supplementary Table 3. Sodium chloride-dependent Msh2-Msh6(3MBD) diffusion coefficients

	**Total Ionic Strength (mM)**	**1D Diffusion ± S.D. (μm^2 s^{-1})**	**Net Displacement (μm)**	**Number of Molecules**	**p-value (relative to 25 mM NaCl)**
**25 mM**	51	(5.8±5.2) x 10^{-2}	0.0±0.9	57	
**50 mM**	76	(5.0±3.7) x 10^{-2}	0.2±1.1	50	4.9 x 10^{-1}
**75mM**	101	(1.2±1.4) x 10^{-1}	-0.2±1.2	58	9.5 x 10^{-4}
**100mM**	126	(1.0±0.9) x 10^{-1}	-0.2±1.2	50	7.1 x 10^{-4}
**150mM**	176	(1.9±1.1) x 10^{-1}	0.1±1.8	54	6.7 x 10^{-16}

Note: All data points were acquired in imaging buffer with 1 mM ADP.

Supplementary Table 4. Oligonucleotides used in this study

Oligo. ID	Sequence
**IF003**	/5Phos/AGG TCG CCG CCC/3BioTEG
**IF004**	/5Phos/GGG CGG CGA CCT/3Dig_N
**MB32**	/5Phos/TGC ATG CGG CCG CTC TTC CCA TGG TGC GAT CGC TCT TCG
**MB34**	/5Phos/TGC ATG CGG CCG CTC TTC CTT TTT TTT TTT TTT TTT T
**MB35**	TGC GAT CGC TCT TCG
**MB36**	/5Phos/TGC ATG CGG CCG CTC TTC CTT TTT TTT TTT TTT TTT T/3Dig_N/
**LS1**	CACGCTACCGAATTCTGACTTGCTAGGACATCTTTGCCCACGTTGACCC
**LS2**	GGGTCAACGTGGGCAAAGATGTCCTAGCAAGTCAGAATTCGGTAGCGTG
**LS6T**	CACGCTACCGAATTCTGACTTGCTAG**T**GACATCTTTGCCCACGTTGACCC
**LS8**	CACGCTACCGAATTCTGACTTGCTAG**GTGTGTGT**GACATCTTTGCCCACGTTGACCC
**LS3**	TCGATAGTCTCTAGATAGCATGTCCTAGCAAGTCAGAATTCGGTAGCGTG
**LS16**	GCTATCTAGAGACTATCGA

Supplementary References

1. Lowary, P. T. & Widom, J. New DNA sequence rules for high affinity binding to histone octamer and sequence-directed nucleosome positioning. *J. Mol. Biol.* **276,** 19–42 (1998).
2. Pathak, S., Davidson, M. C. & Silva, G. A. Characterization of the Functional Binding Properties of Antibody Conjugated Quantum Dots. *Nano Lett.* **7,** 1839–1845 (2007).
3. Dowen, J. M., Putnam, C. D. & Kolodner, R. D. Functional studies and homology modeling of Msh2-Msh3 predict that mispair recognition involves DNA bending and strand separation. *Mol. Cell. Biol.* **30,** 3321–3328 (2010).
